# Research on rice disease recognition based on improved SPPFCSPC-G YOLOv5 network

**DOI:** 10.1371/journal.pone.0295661

**Published:** 2023-12-15

**Authors:** Bo Yang, Lina Zhang, Jinping He

**Affiliations:** 1 School of Information Engineering, Changchun University of Finance and Economics, Changchun, China; 2 School of Information Technology, Jilin Agricultural University, Changchun, China; University of Sindh, PAKISTAN

## Abstract

Spatial Pyramid Pooling (SPP) is important in capturing remote contextual information for pixel-level prediction tasks in scene-resolved detection of rice diseases. In this paper, the detection objects of the rice disease dataset used in this paper have almost the same target size and do not need to be passed through different filters to obtain different receptive fields of view. Therefore, this paper proposed a new pooling structure, SPPFCSPC-G, which split the feature vector into 2 channels for processing. One channel was processed using grouped 1×1 Conv, while the other channel mainly used multiple filters with the same parallel structure (5×5 MaxPool). Additionally, multiple 1×1 and 3×3 grouped convolutions were concatenated in series in that branch (Group-Conv) to extract more complex features in rice. Finally, the 2 parts were connected (Concat) together, with each convolutional layer Conv divided into 4 groups as a way to reduce the amount of computation in the model. The project team incorporated SPPFCSPC-G into the Backbone of YOLOv5 and trained it on NVIDIA Tesla T4 (GPU). The experimental results showed that the performance of the method used in this paper improved, including Precision, Recall, mAP, and training speed, while reducing the size of computational parameters (Parameters), computational volume (GFLOPs), and model size (Param.). The project team carried out the trained YOLOv5 model on Intel Core i5 (CPU) for inference detection of rice leaves in real scenarios, and the experiments showed that both pre-inference and actual inference were faster. Moreover, the consumption of computational resources was almost minimized, and the model effectively identified rice diseases.

## Introduction

Rice, an annual aquatic herbaceous plant in the grass family, is an important cereal crop in tropical Asia. More than half of the world’s population depends on rice as their main food source, and consumer demand for high-quality rice is increasing. However, rice cultivation is often plagued by a variety of diseases [1,2], which severely reduces crop yields and causes significant economic losses [3]. Therefore, the identification and management of rice diseases are essential to ensure sustainable agriculture and food security. Rapid and accurate identification of different types of rice diseases, along with appropriate follow-up solutions and treatment plans, are necessary to prevent further spread and minimize losses.

Detecting objects in an image, as one of the fundamental tasks in today’s agricultural detection and classification related fields, is often used as a starting point for many real-world applications, which include the application of image recognition techniques within the field of computer vision [4–8] to rice leaf pest recognition [4], apple disease recognition [5], citrus fruit recognition [6], plant leaf disease recognition [7,8], and so on.

The widely used deep learning based target detection algorithms can be categorized into two groups:(1) two-step target detection algorithms, such as Fast R-CNN (Region-Convolutional Neural Network) [4], Faster-RCNN [9], MaskR-CNN [10], and so on. These models divide target detection into two phases, firstly using Region Proposal Network (RPN) to extract candidate target region information, then using a classifier for classification, and finally using a detection network to complete the prediction and identification of candidate region categories and locations. (2) Single-step target detection algorithms, such as R-FCN ((Region-based Fully-Convolution Neural Networks) [11], SSD (Single Shot MultiBox Detector) [7], YOLO (You Only Look Once) [12–19]. These algorithms integrate the two tasks of candidate region extraction and classification into a single network, and compute the category scores and positional deviations of the features obtained from the convolutional network directly through a convolutional kernel. The YOLO series, YOLOv1-v3 [12–14], pioneered one-stage object detectors with one-stage, and YOLOv4 [15] categorized the target detection framework as Input, BackBone, Neck, and Head into four independent parts. The subsequent YOLOv5/v6/v7/v8 [16–19] have greatly improved the detection speed and accuracy, and they are all candidates for the deployment of efficient detectors.

SPP module was proposed by KaiMing He et al [20], SPP module effectively avoids the problems of image distortion caused by cropping and scaling operations on image regions, and also solves the problem of repeated feature extraction by CNN for graph correlation, and improves the speed of generating candidate frames. In 2017, Liang-Chieh Chen et al [21], in the semantic segmentation model DeepLabv2 proposed the ASPP module, and they used multiple parallel filters with different rates for multi-scale feature extraction.2018 Songtao Liu et al [22], constructed the RFB module by combining multiple branches with different kernels and an extended Conv. Multiple kernels are analogous to pRFs of different sizes, while the extended convolutional layer assigns a separate eccentricity to each branch to mimic the ratio between the size of the pRF and the eccentricity, while a 1×1 Conv is concatenated in all branches to produce the final feature map.2020 Jocher Glenn [16] proposed SPP-Faster in the open-source YOLOv5 project. In the pooling process he used 2 serially identical small kernel convolutions to replace 1 large kernel, resulting in a parallel structure SPPF, which makes the model much less computationally intensive and the model speed is greatly improved. In 2020 CVPR, Qibin Hou et al [23] changed the convolution to a 1×N or N×1 narrow convolution, thus proposing the concept of Strip Pooling, which inherits the advantages of globally averaged pooling to collect long term dependencies while paying more attention to the local details. In 2022 Chuyi Li et al [17] In YOLOv6, on the basis of SPPF, the activation function SiLU in the convolutional layer is replaced by ReLU, from which SimSPPF is proposed. in 2023CVPR, ChienYao Wang et al [18] proposed the SPPCSPC structure in YOLOv7, which replaces the activation function in the convolutional with ReLU on the basis of the original SPP module or adding the Sigmod function after the convolutional layer, resulting in two new convolutional structures, CMS/CBM, which then capture feature information at different scales through four different fields of view (5, 9, 13MaxPool, 1Conv).

In this study, the SPPF component of the original YOLOv5 structure is modified to SPPFCSPC-G, while the convolutional grouping is introduced, and the superiority of the module is demonstrated through various experiments. Meanwhile the question about whether the performance of the optimizer on home-made datasets is better or worse depends on the theoretical derivation of the computation or on the data itself is explored.

## Data collection and processing

To ensure the inclusiveness and comprehensiveness of the rice disease dataset, three common rice diseases, including leaf blight, brown spot and rice blast, and healthy leaves were selected to construct the dataset in this study. A total of 476 original images were collected with a resolution of 300 pixels × 300 pixels or 300 pixels × 500 pixels.

**Fig 1:**
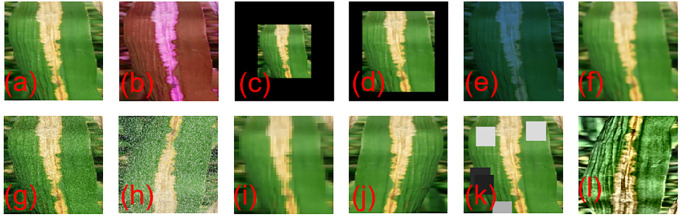
Data enhancement processing. (a) Original Image,(b) HSV,(c)Non-uniform Scaling, (d)Uniform Scaling, (e)Color Temperature Adjustment, (f)Gaussian Blurring,(g) Gaussian noise,(h)Salt-and-pepper noise,(i) Average pooling,(j)Horizontal and vertical flip,(k)Random rectangle occlusion,(l)Adaptive histogram equalization.

To address the temporal and stochastic characteristics of diseased rice leaves in natural environments, collecting complete samples of diseased leaves can be challenging. In order to expand the sample size, improve the accuracy of model training, and prevent overfitting of the convolutional neural network, this study employs data enhancement techniques based on geometric transformations, such as HSV color processing, variable color temperature, Gaussian blur, and pretzel noise. Using data enhancement techniques, the rice pest dataset was doubled and the final training set train was about 800 images. The sample dataset is divided into training set, validation set and test set in the ratio of 8:1:1. Fig 1 shows the image-enhanced leaf blight samples.

## Rice leaf disease identification based on YOLOv5 model

### Construction of YOLOv5 model

**Fig 2:**
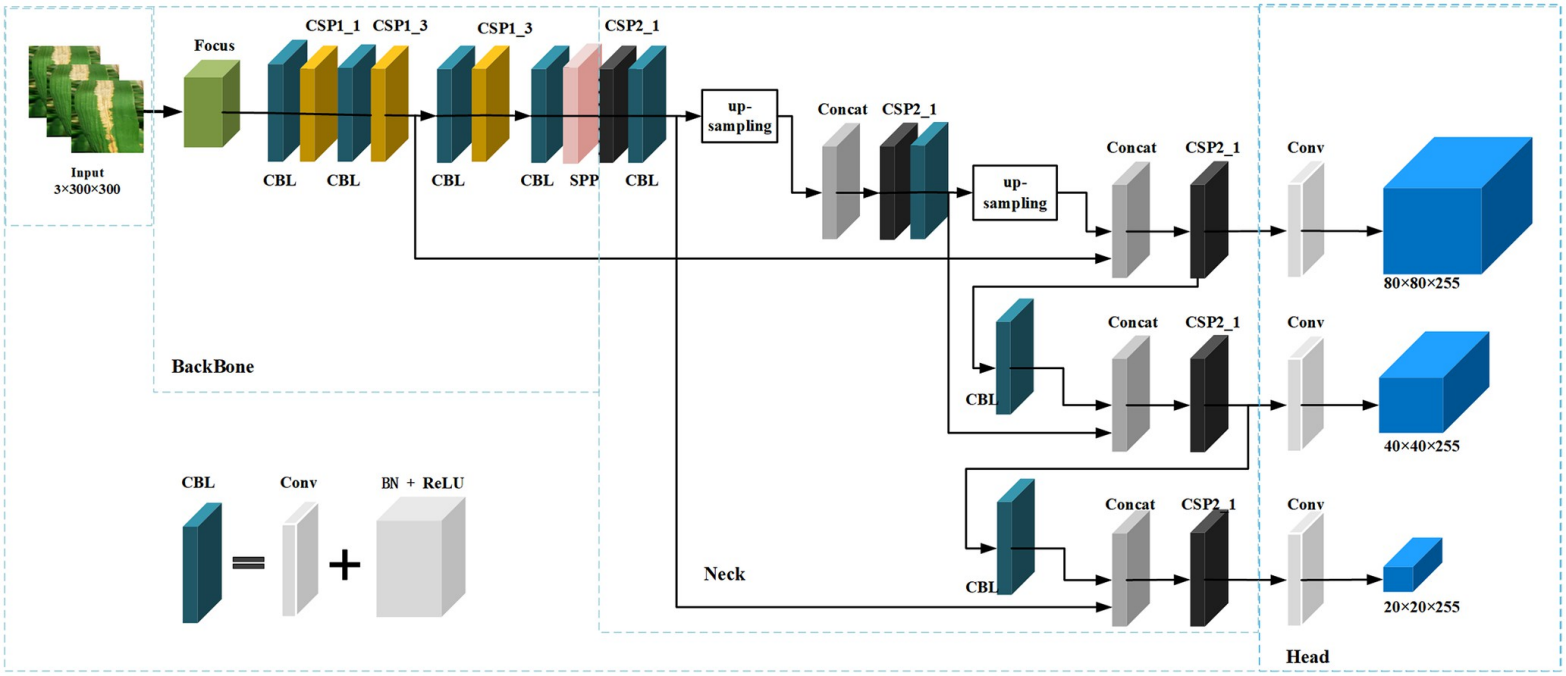
Overall structure diagram of YOLOv5s.

YOLOv5 [16], as a powerful real-time target detection framework inherited from YOLOv4 [15], has good anti-jamming and robustness, which can effectively extract the features of the image as well as learn the feature information implied in the rice disease dataset to achieve better recognition results. The single-level object detector YOLOv5 generally consists of the following four basic parts: Input, BackBone, Neck and Head. Input uses Mosaic data enhancement and proposes an adaptive anchor frame computation and adaptive image scaling method; the main structure in BackBone-CSPDarkNet53 are Focus module, CBL module, C3 module, and SPP module; BackBone determines the feature representation capability and is mainly responsible for feature extraction from the input image; Neck is used for multi-scale feature fusion of low-level physical features with high-level semantic features, and then establishes pyramidal feature mapping at all levels and passes these features to the subsequent prediction end. Head, the prediction end, consists of several convolutional layers and predicts the dynamic detection results based on the multilevel features assembled at the neck for final regression prediction. A typical YOLOv5s is shown in Fig 2.

### Spatial Pyramid Pooling study

#### Classical SPP structure

**SPP** (Spatial Pyramid Pooling) [20], which is equivalent to a standard channel layer. SPP takes an accepted image of any size and extracts spatial feature information of different sizes through a set of standard pooling operations to form a fixed-size feature map, which can be used as an input to the fully connected layer. This improves the robustness of the model to spatial layout and object variability. The core operation of pooling in YOLOv5 is the (5×5, 9×9, 13×13) MaxPool operation. Since SPP is a serial structure, it is pooled only once.

**SPPF** (Spatial Pyramid Pooling Faster) [16] serves the same purpose as SPP except that SPPF is a parallel structure and is given in the following Fig. SPPF replaces the large core structure of 9×9, 13×13 in the original SPP by using two 5×5MaxPool small core structures and connecting three identical 5×5MaxPool components. This results in secondary pooling for richer feature fusion. At the same time, the computation amount of the model becomes much smaller, and the model speed is greatly improved.

**Fig 3:**
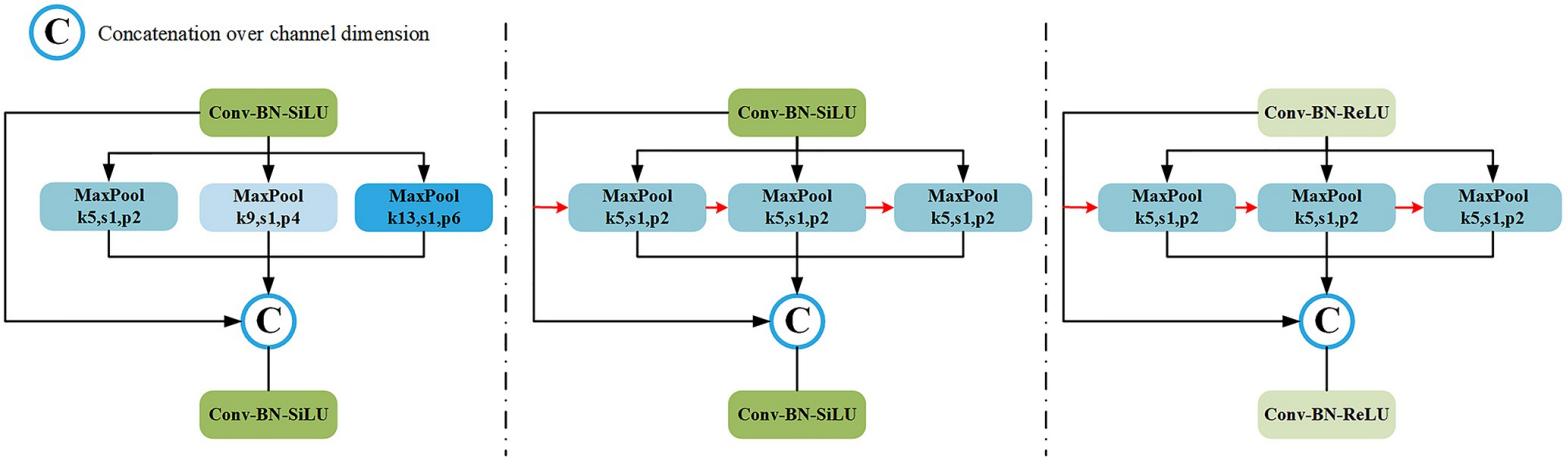
Classic SPP structure image.

**SimSPPF** (Simplified SPPF) [17] only replaces the activation function SiLU of the convolutional layer with ReLU in the SPPF of YOLOv5, and does not make any other adjustments. The structural information of the specific module is shown in Fig 3.

#### Improved SPP structure

**ASPP** (Atrous SPP) [21] is an application of the SPP structure to semantic segmentation, which combines Atrous Convolution to expand the receptive field of the convolution kernel without loss of resolution (no downsampling up-sampling). The essence of ASPP is to use a 1 ×1Conv to firstly perform a 1×1 Input downsampling, and then convolving it with a convolutional layer with padding of 6/12/18, dilation of 6/12/18, and kernel sizes all 3×3. The Input is pooled to 1× 1 with a pooling layer of size Input, then downscaled with a 1×1 convolution, and finally upsampled back to the original input size.

**SPPCSPC** (SPP Cross Stage Partial Channel) [18] is a serial structure that consists of multiple CBS modules connected together with three Maxpool connections. The CBS modules are formed by the Conv layer, the BN layer with the SiLU activation function, and the sensory horizons of the MaxPool are (5×5, 9×9, 13×13).

The SPPFCSPC-G (SPPF Cross Stage Partial Channel-Group) proposed in this paper is composed of multiple grouped convolutional blocks (including 1×1, 3×3), with three serial 5× 5MaxPool layers with the same feeling horizon, connected in parallel with the other components for the secondary pooling operation. Grouped convolution takes the ordinary convolution in component SPPFCSPC-G and performs convolutional grouping by dividing the input feature maps into four groups equally by channel, g = 4. After grouping, the number of channels in each group of the input feature maps Channel is *C*_*in*_/*g*. In each grouping, the number of channels within each convolutional kernel is also reduced to *C*_*in*_/*g*. This results in a drop in the number of operations *FLOPS* = (*C*_*in*_/*g*) × *C*_*out*_ × *O*_*f*_ for the entire grouped convolution over a pixel and the number of parameters for the entire convolution. The channels within each group perform independent convolution operations, and by doing so the computation can be spread across different groups, thus reducing the overall computation. Specific experimental data for paramenters such as FLOPS and parameters can be found in Chapter-Experimental Analysis of Calculated Parameters.

$$Parameters=\left\{\frac{C_{out}\times \left(\frac{C_{in}}{g}\times K_{1}+K_{2}\right),bias=Flase}{C_{out}\times \left(\frac{C_{in}}{g}\times K_{1}+K_{2}+1\right),bias=True}\right.$$ (1)

*O*_*f*_ is the number of operations of a convolutional kernel on top of the complete feature map.

Size of convolution sum is *Kernel* = *K*_1_ × *K*_2_.

**Fig 4:**
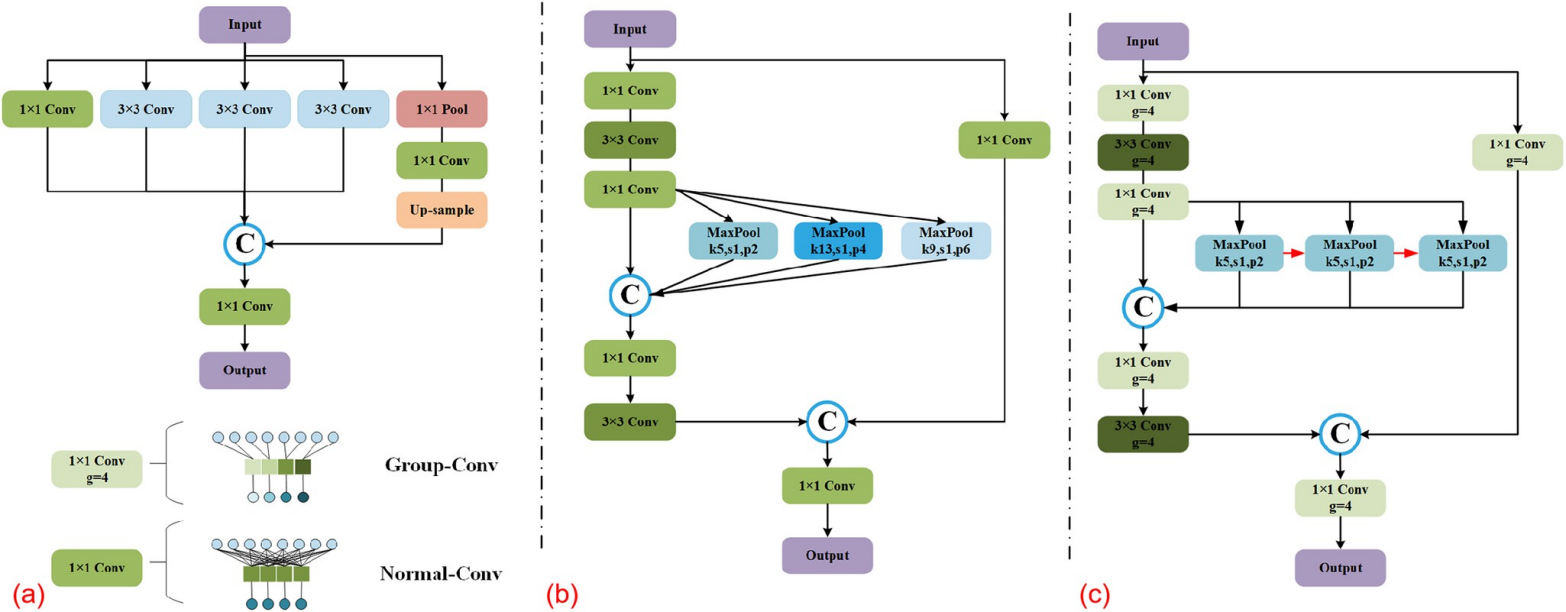
Improved SPP structure image. (a)ASPP,(b)SPPCSPC,(c)SPPFCSPC-G.

Because YOLOv5 contains a large number of convolution and pooling operations, the parallel computing power of GPUs and strong floating-point computing power can quickly complete the computation, reading, and writing of data. Therefore, we train the proposed method on the rice disease dataset using Tesla T4 for each model. SPPFCSPC-G, grouped convolution, and other ideas about the improvement of the SPP module are visualized in Fig 4.

### Optimization

#### Stochastic Gradient Descent (SGD)

SGD [24] goes to update with the same learning rate, and uses gradient descent update for individual samples at each parameter update, only one sample at a time, which does not have to recalculate the gradient as in Batch gradient descent (BGD), which can offset the redundant computation caused by the large dataset, and the training speed is faster. However, this will make the SGD is not every iteration toward the overall optimization, the accuracy will be reduced; the advantage is that in the case of a large number of samples, only part of a few samples can be iterated to the optimal solution, the SGD gradient update rule is as follows:

$$\theta =\theta -\eta .\nabla_{\theta }J(\theta ;x^{\left(i\right)};y^{\left(i\right)})$$ (2)

Training examples *x*^(*i*)^ and labeling examples *y*^(*i*)^ Perform parameter updates.

#### Adaptive Moment Estimation (Adam)

Adaptive Moment Estimation (Adam) [25] is a widely used optimization algorithm that combines the advantages of momentum and adaptive learning rate. It calculates the adaptive learning rate for each parameter by using first- and second-order moment estimation of the gradient, thus dynamically adjusting the learning rate to correct the bias. The computational procedure of Adam can be summarized as follows:

$$m_{t}=\beta_{1}m_{t-1}+(\text{1}-\beta_{1})g_{t}$$ (3)

$$v_{y}=\beta_{2}v_{t-1}+(1-\beta_{2})g_{y}^{2}$$ (4)

When the sum is initialized as a zero vector, it will be biased towards zero, so it is necessary to bias-correct the first and second moment estimates using ${\overset{\wedge }{m}}_{t}=m_{t}/(1-\beta_{1}^{t})$ and ${\overset{\wedge }{v}}_{t}=v_{t}/(1-\beta_{2}^{t})$ to offset these biases. According to the above equation, the parameters are continuously updated to obtain Adam’s gradient update rule as follows:

$$\theta_{t+1}=\theta_{t}-\frac{\eta }{\sqrt{{\overset{\wedge }{v}}_{t}}+\varepsilon }{\overset{\wedge }{m}}_{t}$$ (5)

*m*_*t*_- First-order momentum, the first moment of the gradient, the exponentially shifted mean of the gradient direction at each moment.

*v*_*t*_—second-order moment estimate, an estimate of the uncentered variance of the gradient.

Setting of hyperparameters: first-order momentum decay coefficient *β*_1_ = 0.9; second-order momentum decay coefficient *β*_2_ = 0.999; set equilibrium factor *ε* = 10^−8^ to keep the value stable.

### Loss

The final loss function *L*_*all*_ = *λ*_1_*L*_*cls*_ + *λ*_2_*L*_*box*_ + *λ*_3_*L*_*obj*_ of YOLOv5 mainly consists of a weighted summation of Objectness Loss, Class probability Loss and Bounding box Loss, in which *L*_*box*_ and *L*_*cls*_ are calculated using BCEWithLogits loss, *L*_*box*_ calculates the objective loss of all samples, and *L*_*cls*_ calculates the classification loss of the positive samples only, which is calculated as shown in Eq (6).


$$L_{box}=L_{cls}=\frac{1}{n}\sum_{i}^{n}\left[y_{i}.\text{log}(\sigma (x_{i}))+(1-y_{i}).\text{log}(1-\sigma (x_{i}))\right]$$ (6)

Where the localization loss *L*_*obj*_ is calculated using CIoU loss [26], only the localization loss of positive samples is calculated as shown in Eq (7).


$$L_{obj}=CIoU\_loss=1-IoU(B_{pre},B_{gt})+\frac{\rho^{2}(B_{pre},B_{gt})}{c^{2}}+\alpha v$$ (7)

$$v=\frac{4}{\pi }{\left(\text{arctan}\frac{w_{gt}}{h_{gt}}-\text{arctan}\frac{w_{pre}}{h_{pre}}\right)}^{2}$$ (8)

$$\alpha =\frac{v}{1-IoU(B_{pre},B_{gt})+v}$$ (9)

*λ*_1_*λ*_2_*λ*_3_—weight of the multi-task loss function

*σ*(*x*)—Computational rules for the Sigmoid function *σ*(*x*) = 1/(1 + *e*^−*x*^)

*b*, *w*, *h*—represents the center point, width, height

*pre*, *gt*—represents predicted box and ground truth box

*c*—it is the diagonal distance between the minimum bounding box that encloses both the predicted and true bounding boxes.

*v*—represents the normalized difference between the aspect ratios of the predicted box and the ground truth box.

*α*—Balance factor, Weighting factor to balance the loss caused by aspect ratio and the loss caused by IoU part.

## Experimental results and analysis

### Experimental analysis of SPP

The SPP module was used to connect the BackBone and Neck in the entire network structure of YOLOv5s. Among all the models, most of the YOLOv5s’ BackBones were CSPDarkNet53. The project team also compared the performance of YOLOv5s with Resnet-34/VGG-16 embedded and other SPP modules, as shown in Table 1. From Table 1, it can be seen that the method proposed in this paper performed the best in terms of performance.

**Table 1:** Performance comparison of various SPP structures.

Framework	Model	Optimizer	*SteadyEpoch*	*Precision*	*Recall*	* $mAP_{0.5}^{val}$ *	* $mAP_{0.95}^{val}$ *	Arguments	GPU-mem (G)	Speed-1 forward Propagation (it/s)	Speed-2 backward propagation (it/s)	Speed-3 *AvgEpoch/s*
YOLOv5-s	**SimSPPF**	Adam	326	70.50*%*	71.20*%*	72.50*%*	36.50*%*	[512,512,5]	4.9	1.46	2.05	52.71
	**ASPP**	Adam	315	67*%*	70*%*	71.30*%*	36.40*%*	[512,256]	4.88/4.95	1.13/1.10	1.21/1.22	68.24
	**SPPCSPC**	Adam	339	68.50*%*	70.20*%*	73.30*%*	36.60*%*	[512,512]	5.14/5.24	1.43/1.45	2.02/2.25	49.93
	**SPPFCSPC**	Adam	339	70*%*	71*%*	71.50*%*	36.60*%*	[512,512,5]	5.25/3.86	1.35/6.25	2.37/2.08	—
	**SPPFCSPC-G**(Our)	Adam	300	71*%*	72.3*%*	74.50*%*	37.40*%*	[512,512]	5.14	1.47	2.34	43.70
	Improvement	Adam		**+1.0**	**+1.1**	**+1.2**	**+0.8**					**-12.4*%***
VGG-19	**SPPFCSPC-G**	SGD	278	88.3%	87.70%	89%	78.90%	[512,256,5]	14.7	2.22	1.63	106.43
Resnet-34	**SPPFCSPC**	SGD	264	91.80*%*	93.30*%*	92.10*%*	79.90*%*	[512,512,5]	12.1	1.46	2.07	52.43
Resnet-34	**SPPFCSPC-G**	SGD	267	92.40%	94.45%	93.20%	81%	[512,512,5]	13.9	1.80	2.67	61.83
YOLOv5-s	**SPP**	SGD	248	95*%*	98.13*%*	98.50*%*	86.60*%*	[512,512]	4.91	1.62	2.47	49.47
YOLOv5-s	**SPPF**	SGD	220	95.10*%*	98*%*	98.60*%*	86.70*%*	[512,512,5]	4.91	1.51	2.35	41.91
YOLOv5-s	**SimSPPF**	SGD	222	95.45*%*	98*%*	98.60*%*	85.50*%*	[512,512,5]	4.89	1.45	2.26	44.25
YOLOv5-s	**ASPP**	SGD	183	94.40*%*	98*%*	98*%*	85*%*	[512,256]	—	1.12	1.22	64.24
YOLOv5-s	**SPPCSPC**	SGD	225	95.20*%*	98.10*%*	98.20*%*	86*%*	[512,512]	5.12/5.18	1.48/1.48	2.09/2.26	44.91
YOLOv5-s	**SPPFCSPC**	SGD	209	95.60*%*	98.50*%*	98.50*%*	87*%*	[512,512,5]	5.16	1.43	2.31	—
YOLOv5-s	**SPPFCSPC-G**(Our)	SGD	200	**96.20*%***	**99.22*%***	**98.60*%***	86.80*%*	[512,512]	5.07/3.71	1.42/6.00	2.10	52.71
	Improvement	SGD		**+0.6**	**+0.72**	**=**	**-0.2**					**+5.5*%***

All models were trained based on YOLOv5s architecture. The training for each model was conducted for 400 epochs, except for Resnet/VGG, where the SPP structure was inserted at the 9th layer of the entire network. The batch size was set to 16, and the initial learning rate (lr0) was 0.01, which was decayed by a factor of 0.1 (lrf) during training. A total of 800 images were used for training. The term "SteadyEpoch" represents the initial epoch at which all metrics fully converged, with a maximum increase of 4% in their values.

In the context of the Adam optimizer, the SPPFCSPC-G model achieved superior performance across all parameter metrics, including Precision, Recall, mAP_0.5, and mAP_0.5:0.95, surpassing all other SPP structures. The SPPFCSPC-G model demonstrated significant improvements in all metrics compared to the second-ranked model in each category, with increases of 1.0% for Precision, 1.2% for Recall, 1.2% for mAP_0.5, and 0.8% for mAP_0.5:0.95. Moreover, the SPPFCSPC-G model exhibited an average training speed 12.4% faster than the second-ranked SPPCSPC model. This means that SPPFCSPC-G only required 43.70 seconds to complete the entire process of initializing parameters, forward propagation, loss computation, backward propagation, and parameter updates for 800 images.

In the SGD optimizer, the SPPFCSPC-G model also demonstrated the best Precision and Recall metrics, eventually converging to around 96.20% and 99.22%, respectively. The mAP_0.5 metric did not show significant differences among the nine models. However, mAP_0.5:0.95 decreased by 0.2%. Nevertheless, when considering other parameters, such as Loss, F1_Score, Confusion-Matrix, Param., Parameters, GFLOPs, and the actual detection performance, the decrease in training speed could be considered negligible. Detailed results were provided in this subsequent section.

### Experimental analysis of optimizer

#### Comparison of recognition precision performance

Adam, as an amalgamation of SGD-M and RMSprop, combined the advantages of momentum and adaptive learning rate, making it an optimization method that calculates an adaptive learning rate for each parameter. The theoretical calculations suggested that it should outperform SGD. However, the performance of optimizers on a dataset depends not only on theoretical derivations but also significantly on the characteristics of the data itself. By combining Table 2 and Table 1, it was evident that on the rice pest dataset, SGD significantly outperformed Adam on various parameter metrics (Precision, Recall, mAP_0.5), improving by over 25% in nearly all cases. Moreover, the difference in mAP_0.5:0.95 reached 50%. This indicates that, in specific scenarios, the characteristics of the data can play a crucial role in determining the performance of the optimizer.

**Table 2:** Comparison of recognition precision performance of four objects in SGD and Adam.

Optimizer	Class	*Precision*	*Recall*	$mAP_{0.5}^{val}$	$mAP_{0.95}^{val}$
**SGD**	0: blast	99.20%	99.12%	99.50%	89.10%
	1: blight	82.30%	97.50%	96.10%	78.40%
	2: healthy	99.50%	98.70%	99.50%	92.80%
	3: spot	99.20%	99%	99.50%	86.80%
**Adam**	0: blast	61.80%	85.60%	79.40%	32.90%
	1: blight	61.90%	36.60%	50.70%	17.20%
	2: healthy	97.20%	86.40%	96%	77%
	3: spot	59.40%	78.50%	73%	24.20%
**Adam**	All	70%	71%	73%	36.60%
**SGD**	All	95.50%	98%	98%	86.60%
Improvement		**+25.5**	**+27**	**+25**	**+50**

#### F1_Score and Confusion Matrix

F-score is a commonly used metric for evaluating the precision of an algorithm, which provides a comprehensive performance evaluation metric that reflects the performance of an algorithm in a balanced way by considering both precision P and recall R, and by calculating the reconciled average of the two. Its calculation principle is *F*_1__*Score* = 2*PR*/(*P* + *R*).

Confusion Matrix is a matrix table intuitively used for evaluating the performance of a classification model for visualizing and summarizing the predictions of a classifier, which compares the true labels from the rice disease leaf test set test with the predictions of the model and displays them in a matrix. The rows of the confusion matrix represent the true labels and the columns represent the model predictions. The number in each cell of the main diagonal represents the intersection of the true label and the model prediction result.

By examining the trends of all the data presented below, it became evident that the performance of the SGD optimizer outperformed the Adam optimizer on the dataset used in this study.

Among the metrics considered were Precision, Recall, P-R curve, F1_Score, and Confusion-Matrix, and the performance of the SGD optimizer consistently outperformed the Adam optimizer on the dataset used in this study.

### Experimental analysis of the loss function

**Fig 5:**
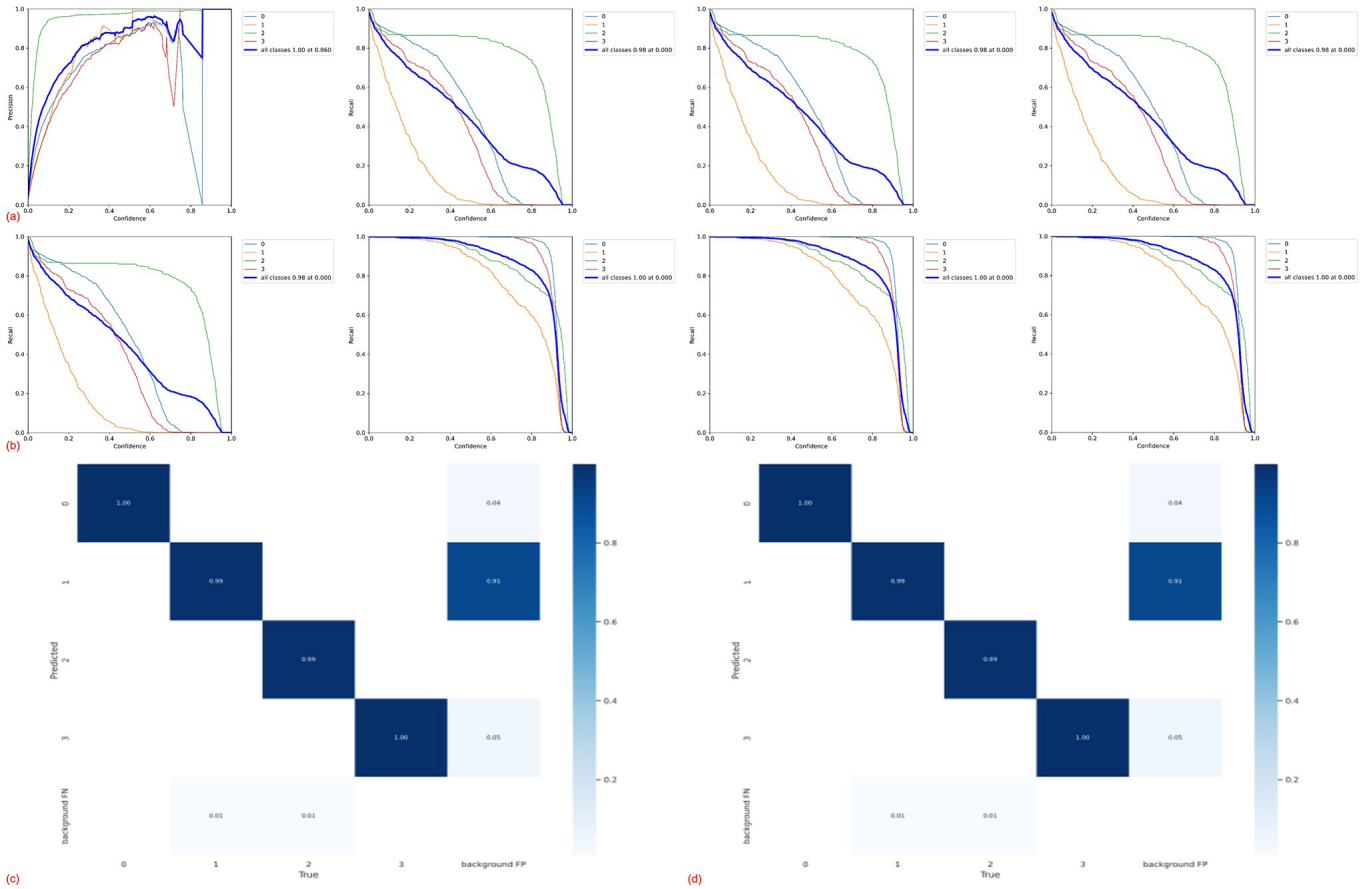
Details of model training of YOLOv5s-SPPCSPC-G on the dataset. (a)SPPCSPC-G_Adam,(b)SPPCSPC-G_SGD,(c)SPPCSPC-G_Adam,(d)SPPCSPC-G_SGD.

Loss is used to measure the difference between the true and predicted values between models, and to some extent determines the merit of the model. The training process of the entire experiment was iterated for 400 epochs. To ensure the stability of the model, the warm-up learning strategy was employed for the first 30 epochs, setting the initial learning rate (lr0) to 0.01. Subsequently, the cosine annealing algorithm controlled the decay of the learning rate, with a hyperparameter of lrf = 0.1, and the minimum learning rate was set to 0.001. After 400 epochs of training, the loss curve obtained is shown in Fig 5. In this Fig, box_loss represents the magnitude of the difference between the model-predicted bounding box and the true bounding box (GIoU), cls_loss is the classification loss used to calculate whether the anchor box is correctly classified with the corresponding calibration, and obj_loss is the confidence loss, which supervises the presence of Grid objects and calculates the confidence level of the network.

**Fig 6:**
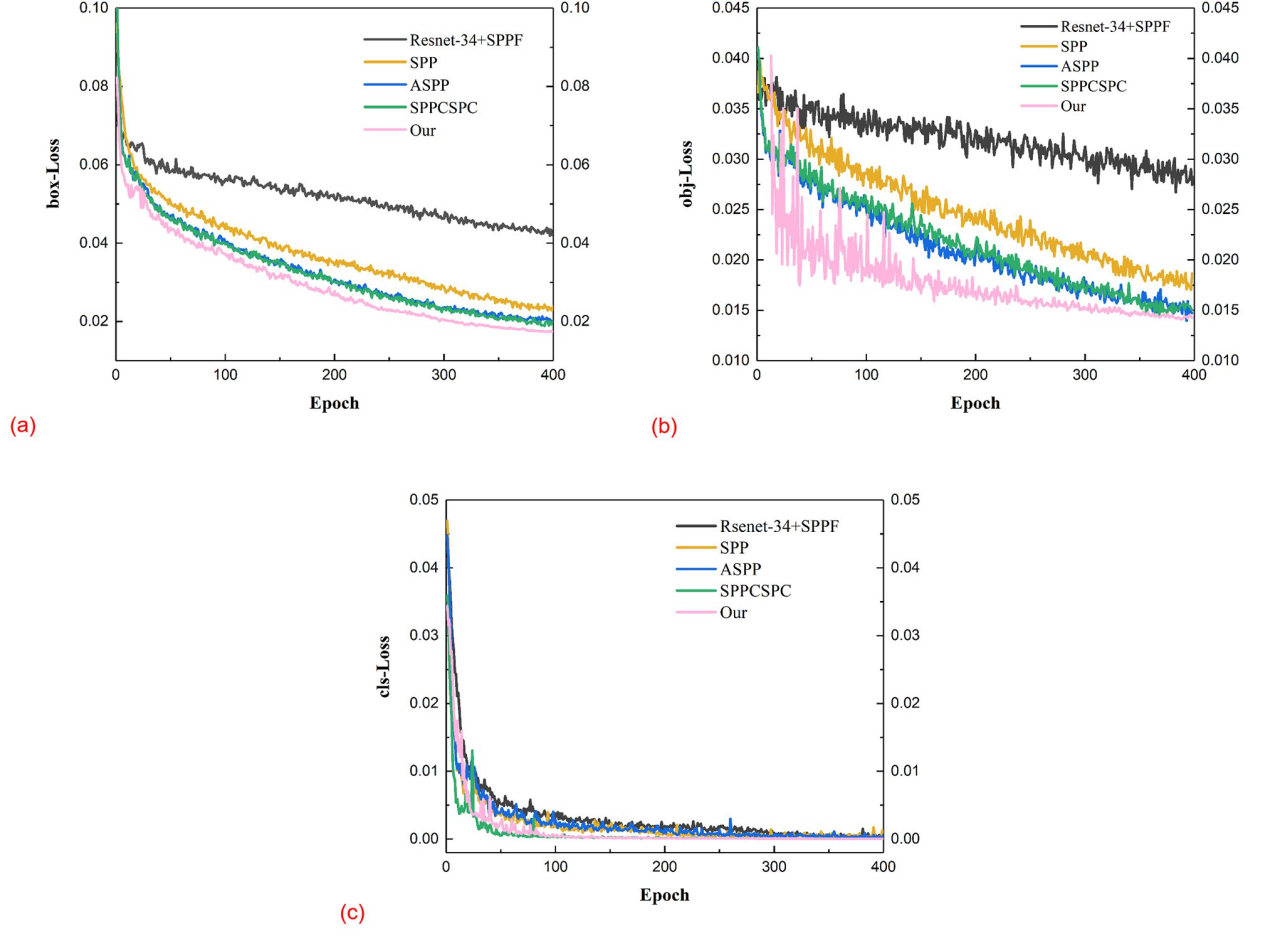
Comparison of loss functions on the training set. (a)bounding box Loss, (b)objectness Loss,(c)class probability Loss.

As can be seen in Fig 6, when the epoch is 300/200/100 rounds, the Bounding box Loss, Objectness Loss, and Class probability Loss values of the YOLOv5s-SPPFCSPC-G network have stopped decreasing, reached the convergence point, and stabilized. This indicates that the SPPFCSPC-G model has learned an optimal solution on the given rice diseased leaf training data, and the model has successfully captured the important features in the rice diseased leaf dataset, and thus learned the nature of the data. Also on the TRAIN our SPPFCSPC-G model has the best convergence.

### Experimental analysis of computational parameters

#### Training

Considering that this work will be applied to mobile detection in the future, it provides detection models for industrialized real-world applications. We focus primarily on the complexity and computation of all models after deployment and secondarily on the speed of the models.

In YOLOv5s, we use different optimizers (SGD, Adam) and the same learning scheme (warm-up warm-up learning strategy, cosine annealing algorithm) to train the designed models separately. All our models are trained on 1 NVIDIA Tesla T4 graphics processor and the speed performance is also measured on NVIDIA Tesla T4 graphics processor, the training results are shown in Table 3.

**Table 3:** Parameters when training the model using Tesla T4.

Framework	Model	Layers	Group	Parameters	GFLOPs (G)	Params. (M)
Resnet-34	**SPPFCSPC_G**	235	***×***	24143633	62.4	45.3
VGG-19	**SPPFCSPC_G**	160	***×***	17655569	50.6	38.8
YOLOv5-s	**SPP**	273	***×***	7030417	16.6	14.8
	**SPPF**	270	***×***	7030417	16.0	14.8
	**SimSPPF**	270	***×***	7030417	16.6	14.8
	**ASPP**	268	***×***	10438289	19.3	20.1
	**SPPCSPC**	293	***×***	13458577	21.8	25.9
	**SPPFCSPC**	290	***×***	13458577	21.1	25.9
	**SPPFCSPC_G**	**293**	**√**, **g = 4**	**8150161**	**16.9**	**15.8**

Note: Except for Resnet/VGG where only SGD was used, all models were trained separately once each using Adam, SGD optimizers, epoch = 400. the training results (Parameters, GFLOPs, Params., Layers) for the same model, under different optimizers, are identical.

SPPFCSPC_G after convolutional grouping and our optimization strategy has its Parameters, which are only 15.9% higher than SPP/SPPF/SimSPPF with 270 layers, but 65.1% lower than SPPCSPC/SPPFCSPC with the same number of layers, for a network structure with a depth of 293 layers. GFLOPs are the GFLOPs that are used in performing the number of floating-point operations (in billions of floating-point operations) required during the forward propagation of the model, and larger values of GFLOPs indicate that the model needs to perform more floating-point operations, and thus may require longer inference times. Despite the reduced parameters of our model, SPPFCSPC_G is the best performing model in the previous SPP experimental analysis chapters, which indicates that the expressive power of our model does not decrease as a result.

Also, the GFLOPs operation of our model is almost minimized, and the final SPPFCSPC_G model is only 15.8M for the actual model trained in Adam/SGD.

#### Reasoning

Various models trained with Intel(R) Core-i5 were used to perform rice disease leaf image detection inference in YOLOv5s and the related data can be viewed in Table 4. All the models were trained with 400 epochs without pre-training or any external data. Both the accuracy and speed performance of the models were evaluated at an input resolution of 640 × 640.

**Table 4:** Inferred Data (in Intel(R) Core-i5 hardware environment).

Model	Optimizer	InputSize	Layers	Parameters	Preprocessing (ms)	Reasoning (ms)	*NMS* (ms)	GFLOPs (G)
**SPP**	SGD	640	216	7020913	15.5	388.8	1.0	15.8
**SPPF**	SGD	640	213	7020913	4.4	353.2	4.0	15.8
**SimSPPF**	SGD	640	215	7021681	3.5	333.2	1.2	15.8
	Adam	640	215	7021681	10.0	416.7	1.0	15.8
**ASPP**	SGD	640	213	10429553	3.7	339.5	3.5	18.5
	Adam	640	213	10429553	4.2	349.7	0.7	18.5
**SPPCSPC**	SGD	640	231	13446257	3.5	458.7	1.0	20.9
	Adam	640	231	13446257	3.2	441.0	1.0	20.9
**SPPFCSPC**	SGD	640	228	13446257	3.2	389.4	0.7	20.9
	Adam	640	228	13446257	3.0	397.9	1.3	20.9
**SPPFCSPC_G**	SGD	640	231	8137841	3.2	452.5	1.0	16.7
	Adam	640	231	8137841	5.2	444.3	1.0	16.7

In this study, the project team used the trained SPPFCSPC-G_Adam and SPPFCSPC-G_SGD models to reason on a test dataset containing approximately 100 image samples in an Intel(R) Core(TM) i5 hardware environment to identify four classes of samples, including healthy, blight, spot, blast. the model provides class confidence for each recognized image, with a preprocessing speed of 3.2ms/image and a recognition speed of 0.44s/image for a single image with NMS = 1, but this is not the fastest speed. In all the previous experimental chapters, the performance of SPPFCSPC-G outperforms the other models in all other aspects, and because of this, the performance improvement that this model brings, the resulting speed reduction, is negligible.

**Fig 7:**
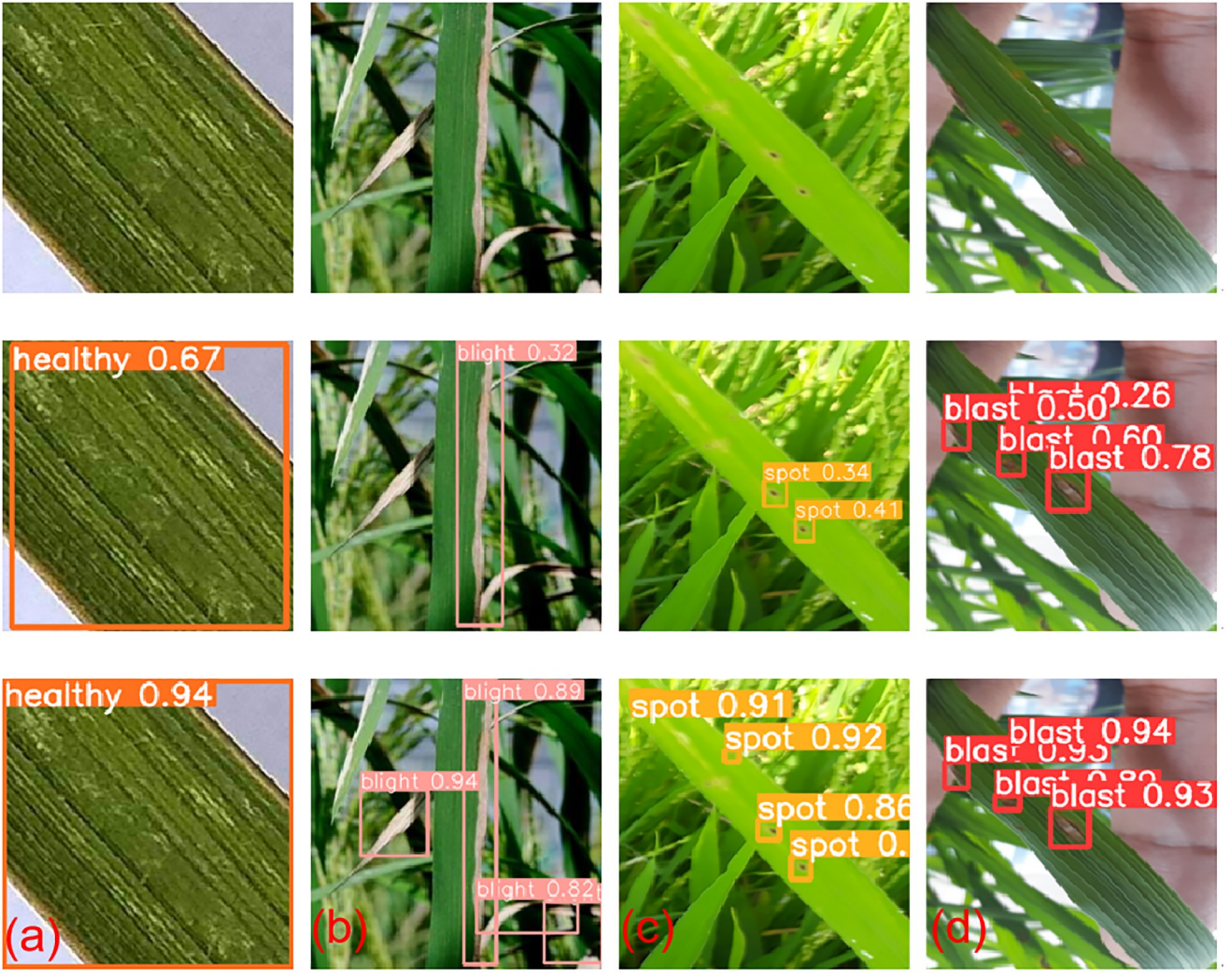
Identification effect drawing. (a)Healthy state,(b)Bacterial leaf blight,(c)Brown spot disease,(d)Rice blast disease.

As shown in Fig 7, the project team put the models trained by SPPFCSPC_G under Adam/SGD to reason about the detection of rice leaves in real scenarios, respectively. adam only detected more obvious target objects in disease detection of blight and spot, and the actual detection effect for small targets and dark objects was not ideal, while the class confidence of each image is also lower, but the detection effect using SGD is better.

## Conclusion

This study presents a novel approach for recognizing and detecting rice leaf diseases in natural environments using the improved YOLOv5-SPPFCSPC-G model. Initially, a series of data augmentation techniques were applied to the original rice images to expand the dataset. Next, the structure and enhancement methods of various SPP modules were reviewed. The new SPPFCSPC-G module was then designed by combining the structure of convolutional grouping and SPPCSPC, and embedded into the BackBone of YOLOv5s.

Thirteen different models were trained and the superiority of the SPPFCSPC-G model was demonstrated through various experiments, including SPP experiments, optimizer experiments, Loss experiments, and computational parameter experiments. The results were analyzed and compared from different perspectives. Furthermore, this paper explored the question of whether the optimizer’s performance on the dataset depends on the theoretical derivation of the computation or on the characteristics of the data itself.

Finally, the trained YOLOv5 model was applied for actual detection, and it showed good detection results. This approach effectively assisted in the detection and identification of rice diseases.

## Supporting information

S1 Data (ZIP)
