# Supplementary figures and images for: Research on rice disease recognition based on improved SPPFCSPC-G YOLOv5 network (part 2 of 2)
Source: PLoS One. 2023 Dec 15;18(12):e0295661. doi: 10.1371/journal.pone.0295661 (PMC10723668; doi:10.1371/journal.pone.0295661)

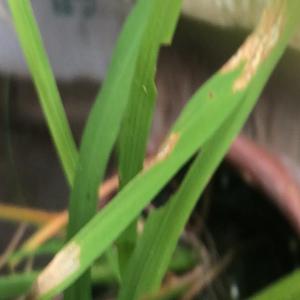

Supplement: S1 Data — (ZIP) [file pone.0295661.s001.zip › rice_images/blast/images/blast_rotated_021.jpg]

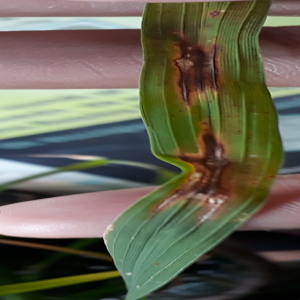

Supplement: S1 Data — (ZIP) [file pone.0295661.s001.zip › rice_images/blast/images/blast_rotated_022.png]

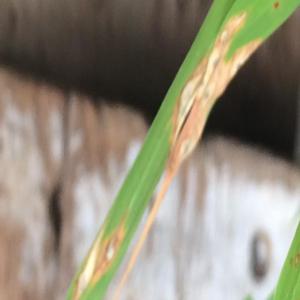

Supplement: S1 Data — (ZIP) [file pone.0295661.s001.zip › rice_images/blast/images/blast_rotated_023.jpg]

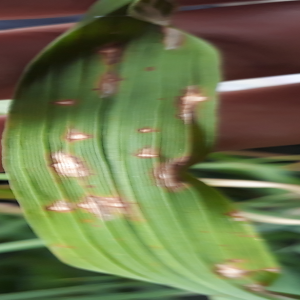

Supplement: S1 Data — (ZIP) [file pone.0295661.s001.zip › rice_images/blast/images/blast_rotated_024.png]

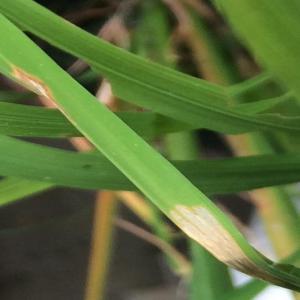

Supplement: S1 Data — (ZIP) [file pone.0295661.s001.zip › rice_images/blast/images/blast_rotated_025.jpg]

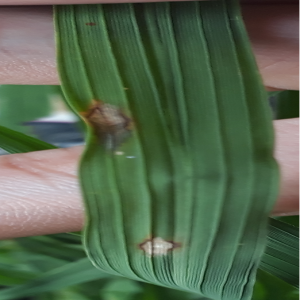

Supplement: S1 Data — (ZIP) [file pone.0295661.s001.zip › rice_images/blast/images/blast_rotated_026.png]

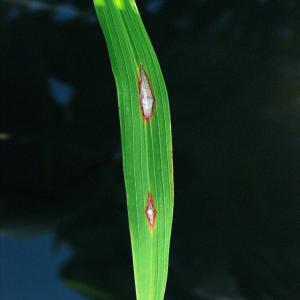

Supplement: S1 Data — (ZIP) [file pone.0295661.s001.zip › rice_images/blast/images/blast_rotated_027.jpg]

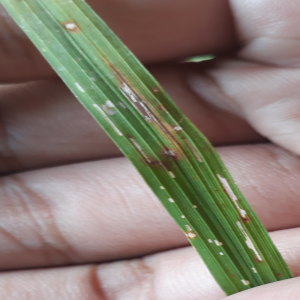

Supplement: S1 Data — (ZIP) [file pone.0295661.s001.zip › rice_images/blast/images/blast_rotated_028.png]

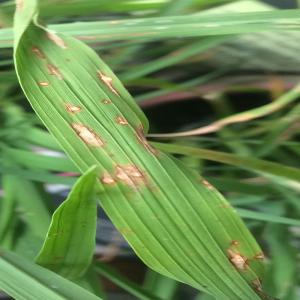

Supplement: S1 Data — (ZIP) [file pone.0295661.s001.zip › rice_images/blast/images/blast_rotated_029.JPG]

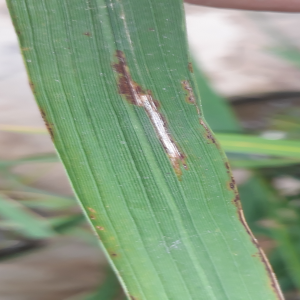

Supplement: S1 Data — (ZIP) [file pone.0295661.s001.zip › rice_images/blast/images/blast_rotated_030.png]

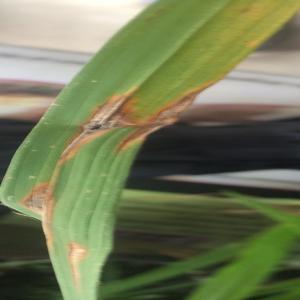

Supplement: S1 Data — (ZIP) [file pone.0295661.s001.zip › rice_images/blast/images/blast_rotated_031.JPG]

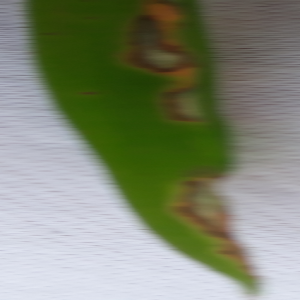

Supplement: S1 Data — (ZIP) [file pone.0295661.s001.zip › rice_images/blast/images/blast_rotated_032.png]

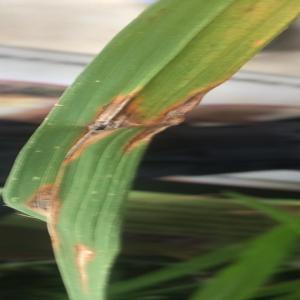

Supplement: S1 Data — (ZIP) [file pone.0295661.s001.zip › rice_images/blast/images/blast_rotated_033.JPG]

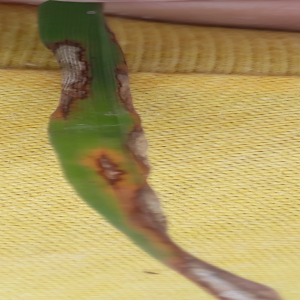

Supplement: S1 Data — (ZIP) [file pone.0295661.s001.zip › rice_images/blast/images/blast_rotated_034.png]

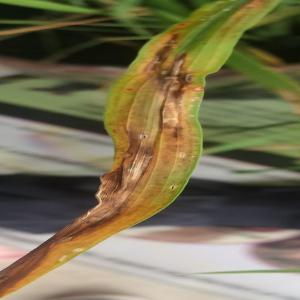

Supplement: S1 Data — (ZIP) [file pone.0295661.s001.zip › rice_images/blast/images/blast_rotated_035.JPG]

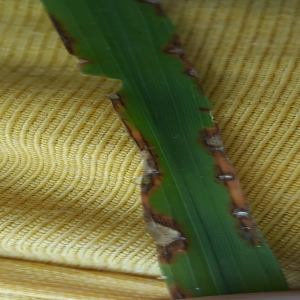

Supplement: S1 Data — (ZIP) [file pone.0295661.s001.zip › rice_images/blast/images/blast_rotated_036.png]

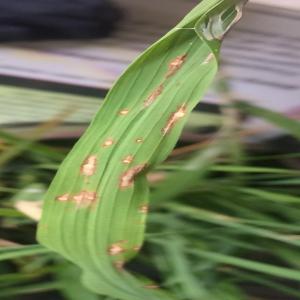

Supplement: S1 Data — (ZIP) [file pone.0295661.s001.zip › rice_images/blast/images/blast_rotated_037.JPG]

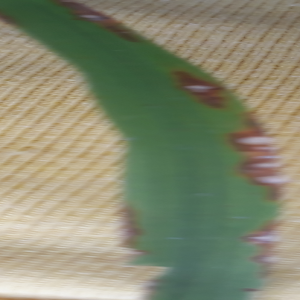

Supplement: S1 Data — (ZIP) [file pone.0295661.s001.zip › rice_images/blast/images/blast_rotated_038.png]

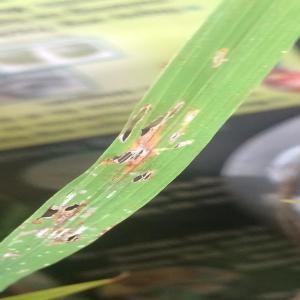

Supplement: S1 Data — (ZIP) [file pone.0295661.s001.zip › rice_images/blast/images/blast_rotated_039.JPG]

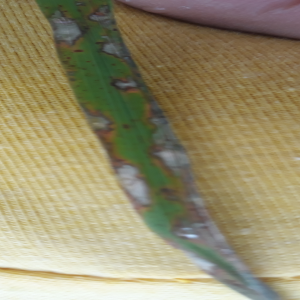

Supplement: S1 Data — (ZIP) [file pone.0295661.s001.zip › rice_images/blast/images/blast_rotated_040.png]

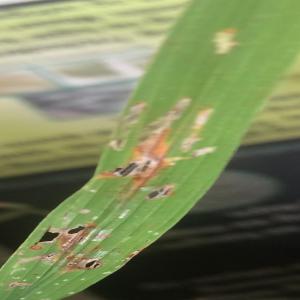

Supplement: S1 Data — (ZIP) [file pone.0295661.s001.zip › rice_images/blast/images/blast_rotated_041.JPG]

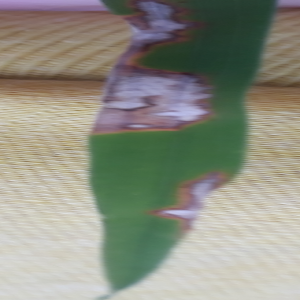

Supplement: S1 Data — (ZIP) [file pone.0295661.s001.zip › rice_images/blast/images/blast_rotated_042.png]

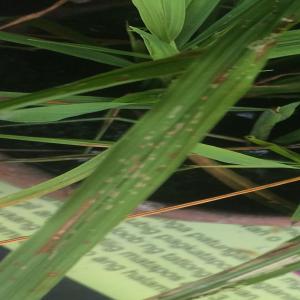

Supplement: S1 Data — (ZIP) [file pone.0295661.s001.zip › rice_images/blast/images/blast_rotated_043.JPG]

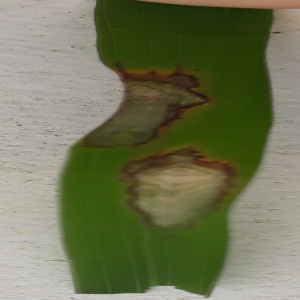

Supplement: S1 Data — (ZIP) [file pone.0295661.s001.zip › rice_images/blast/images/blast_rotated_044.png]

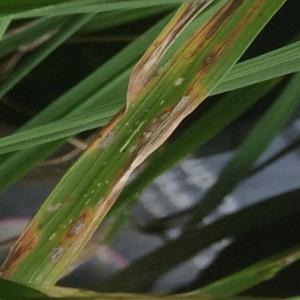

Supplement: S1 Data — (ZIP) [file pone.0295661.s001.zip › rice_images/blast/images/blast_rotated_045.jpg]

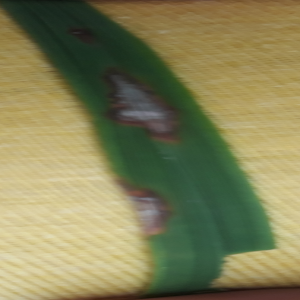

Supplement: S1 Data — (ZIP) [file pone.0295661.s001.zip › rice_images/blast/images/blast_rotated_046.png]

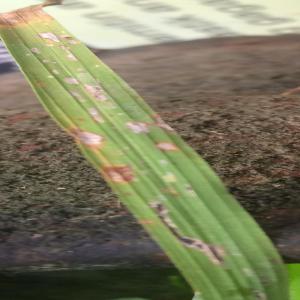

Supplement: S1 Data — (ZIP) [file pone.0295661.s001.zip › rice_images/blast/images/blast_rotated_047.JPG]

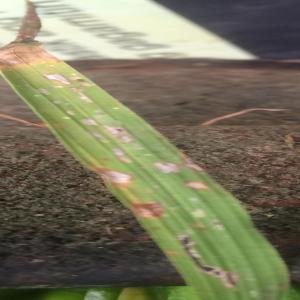

Supplement: S1 Data — (ZIP) [file pone.0295661.s001.zip › rice_images/blast/images/blast_rotated_048.JPG]

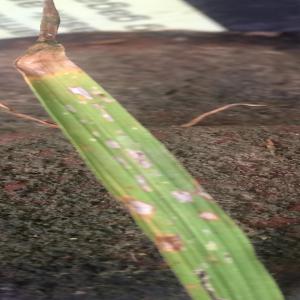

Supplement: S1 Data — (ZIP) [file pone.0295661.s001.zip › rice_images/blast/images/blast_rotated_049.JPG]

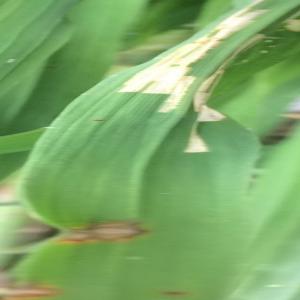

Supplement: S1 Data — (ZIP) [file pone.0295661.s001.zip › rice_images/blast/images/blast_rotated_050.JPG]

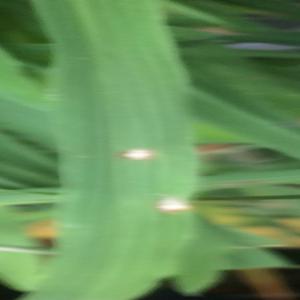

Supplement: S1 Data — (ZIP) [file pone.0295661.s001.zip › rice_images/blast/images/blast_rotated_051.jpg]

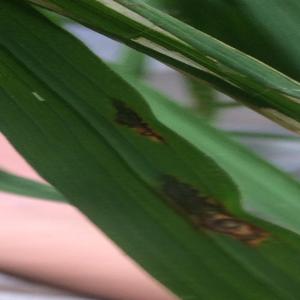

Supplement: S1 Data — (ZIP) [file pone.0295661.s001.zip › rice_images/blast/images/blast_rotated_052.JPG]

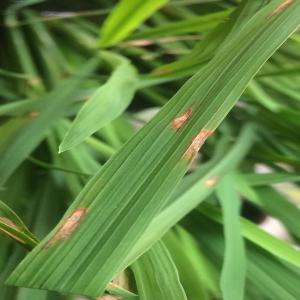

Supplement: S1 Data — (ZIP) [file pone.0295661.s001.zip › rice_images/blast/images/blast_rotated_053.JPG]

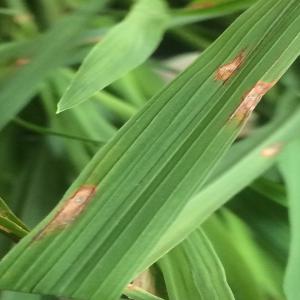

Supplement: S1 Data — (ZIP) [file pone.0295661.s001.zip › rice_images/blast/images/blast_rotated_054.jpg]

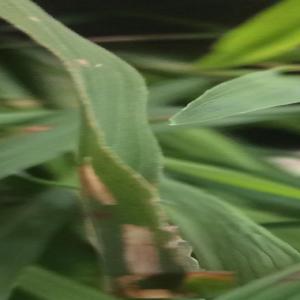

Supplement: S1 Data — (ZIP) [file pone.0295661.s001.zip › rice_images/blast/images/blast_rotated_055.JPG]

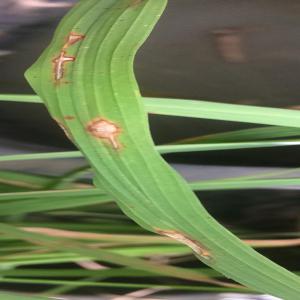

Supplement: S1 Data — (ZIP) [file pone.0295661.s001.zip › rice_images/blast/images/blast_rotated_056.JPG]

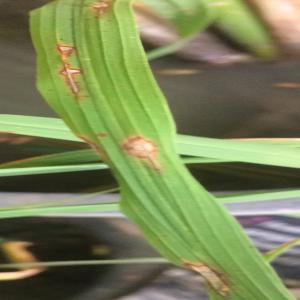

Supplement: S1 Data — (ZIP) [file pone.0295661.s001.zip › rice_images/blast/images/blast_rotated_057.JPG]

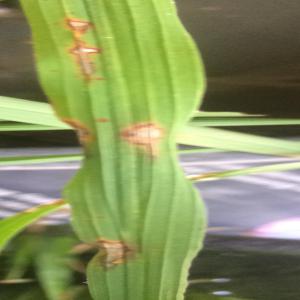

Supplement: S1 Data — (ZIP) [file pone.0295661.s001.zip › rice_images/blast/images/blast_rotated_058.JPG]

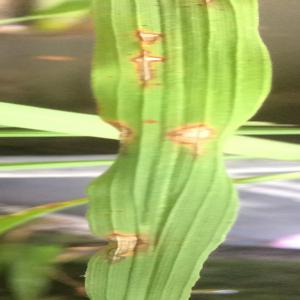

Supplement: S1 Data — (ZIP) [file pone.0295661.s001.zip › rice_images/blast/images/blast_rotated_059.JPG]

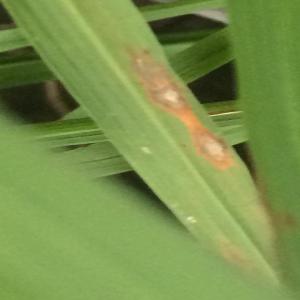

Supplement: S1 Data — (ZIP) [file pone.0295661.s001.zip › rice_images/blast/images/blast_rotated_060.JPG]

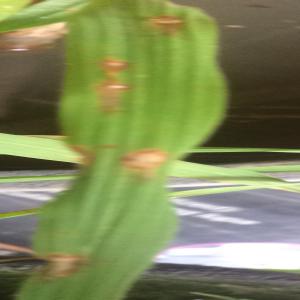

Supplement: S1 Data — (ZIP) [file pone.0295661.s001.zip › rice_images/blast/images/blast_rotated_061.jpg]

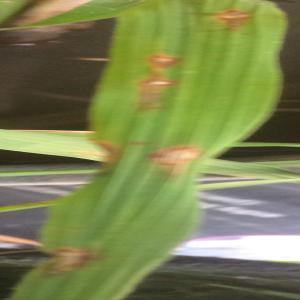

Supplement: S1 Data — (ZIP) [file pone.0295661.s001.zip › rice_images/blast/images/blast_rotated_062.JPG]

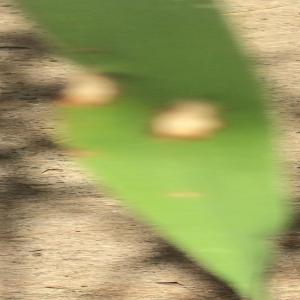

Supplement: S1 Data — (ZIP) [file pone.0295661.s001.zip › rice_images/blast/images/blast_rotated_063.JPG]

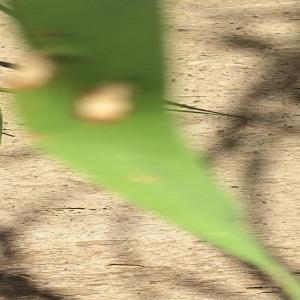

Supplement: S1 Data — (ZIP) [file pone.0295661.s001.zip › rice_images/blast/images/blast_rotated_064.JPG]

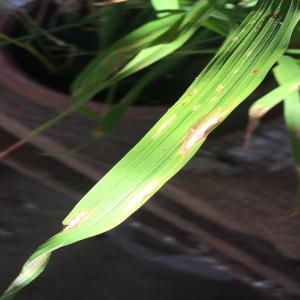

Supplement: S1 Data — (ZIP) [file pone.0295661.s001.zip › rice_images/blast/images/blast_rotated_065.JPG]

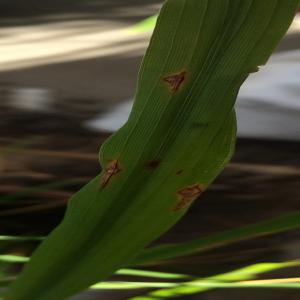

Supplement: S1 Data — (ZIP) [file pone.0295661.s001.zip › rice_images/blast/images/blast_rotated_066.JPG]

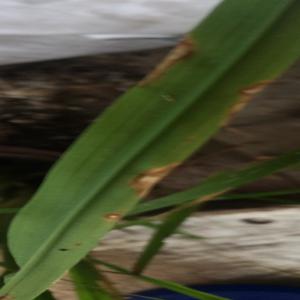

Supplement: S1 Data — (ZIP) [file pone.0295661.s001.zip › rice_images/blast/images/blast_rotated_067.JPG]

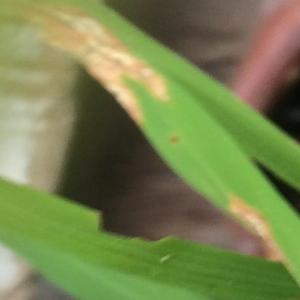

Supplement: S1 Data — (ZIP) [file pone.0295661.s001.zip › rice_images/blast/images/blast_rotated_068.JPG]

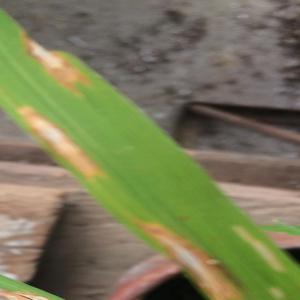

Supplement: S1 Data — (ZIP) [file pone.0295661.s001.zip › rice_images/blast/images/blast_rotated_069.JPG]

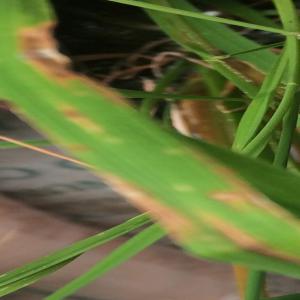

Supplement: S1 Data — (ZIP) [file pone.0295661.s001.zip › rice_images/blast/images/blast_rotated_070.JPG]

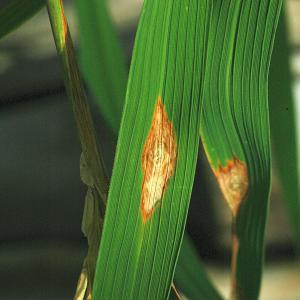

Supplement: S1 Data — (ZIP) [file pone.0295661.s001.zip › rice_images/blast/images/blast_rotated_071.jpg]

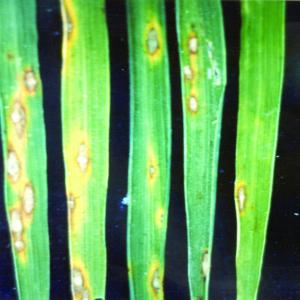

Supplement: S1 Data — (ZIP) [file pone.0295661.s001.zip › rice_images/blast/images/blast_rotated_072.jpg]

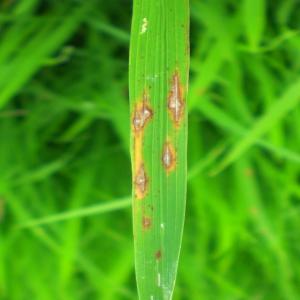

Supplement: S1 Data — (ZIP) [file pone.0295661.s001.zip › rice_images/blast/images/blast_rotated_073.jpg]

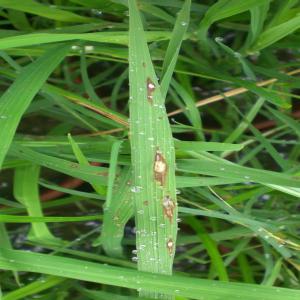

Supplement: S1 Data — (ZIP) [file pone.0295661.s001.zip › rice_images/blast/images/blast_rotated_074.jpg]

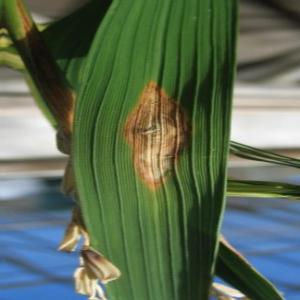

Supplement: S1 Data — (ZIP) [file pone.0295661.s001.zip › rice_images/blast/images/blast_rotated_075.jpg]

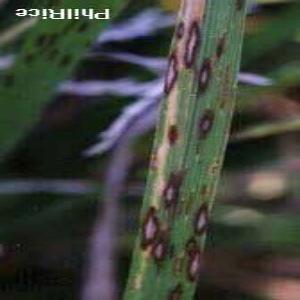

Supplement: S1 Data — (ZIP) [file pone.0295661.s001.zip › rice_images/blast/images/blast_rotated_076.jpg]

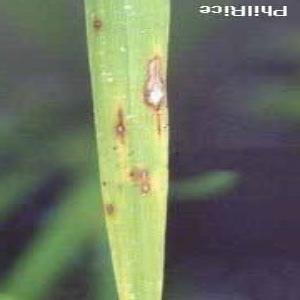

Supplement: S1 Data — (ZIP) [file pone.0295661.s001.zip › rice_images/blast/images/blast_rotated_077.jpg]

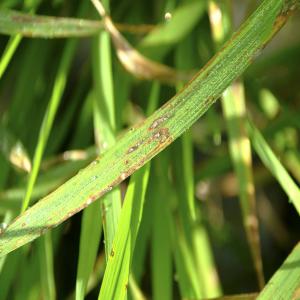

Supplement: S1 Data — (ZIP) [file pone.0295661.s001.zip › rice_images/blast/images/blast_rotated_078.jpg]

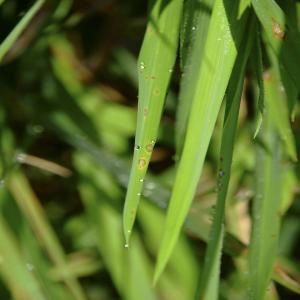

Supplement: S1 Data — (ZIP) [file pone.0295661.s001.zip › rice_images/blast/images/blast_rotated_079.jpg]

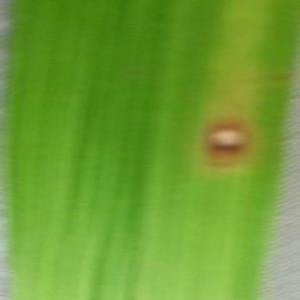

Supplement: S1 Data — (ZIP) [file pone.0295661.s001.zip › rice_images/blast/images/blast_rotated_080.jpg]
