# Supplementary figures and images for: Research on rice disease recognition based on improved SPPFCSPC-G YOLOv5 network (part 1 of 2)
Source: PLoS One. 2023 Dec 15;18(12):e0295661. doi: 10.1371/journal.pone.0295661 (PMC10723668; doi:10.1371/journal.pone.0295661)

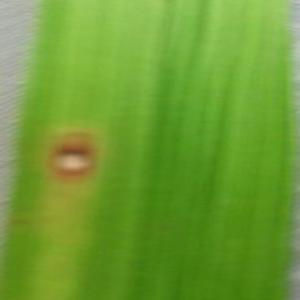

Supplement: S1 Data — (ZIP) [file pone.0295661.s001.zip › rice_images/blast/images/blast_orig_001.jpg]

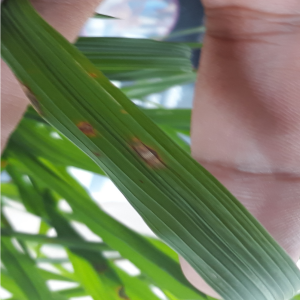

Supplement: S1 Data — (ZIP) [file pone.0295661.s001.zip › rice_images/blast/images/blast_orig_002.png]

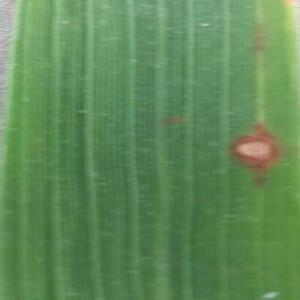

Supplement: S1 Data — (ZIP) [file pone.0295661.s001.zip › rice_images/blast/images/blast_orig_003.jpg]

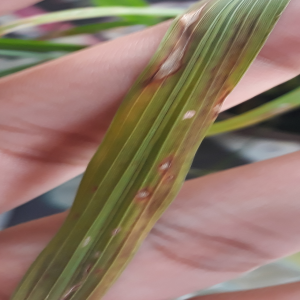

Supplement: S1 Data — (ZIP) [file pone.0295661.s001.zip › rice_images/blast/images/blast_orig_004.png]

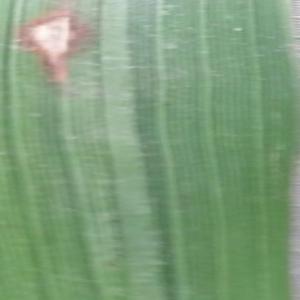

Supplement: S1 Data — (ZIP) [file pone.0295661.s001.zip › rice_images/blast/images/blast_orig_005.jpg]

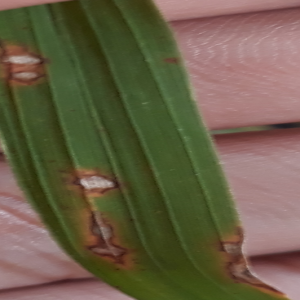

Supplement: S1 Data — (ZIP) [file pone.0295661.s001.zip › rice_images/blast/images/blast_orig_006.png]

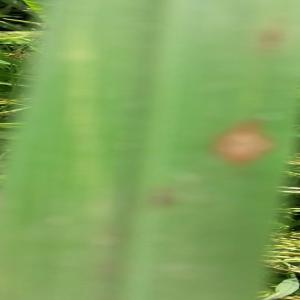

Supplement: S1 Data — (ZIP) [file pone.0295661.s001.zip › rice_images/blast/images/blast_orig_007.jpg]

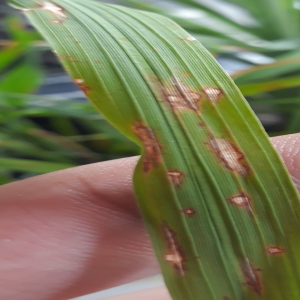

Supplement: S1 Data — (ZIP) [file pone.0295661.s001.zip › rice_images/blast/images/blast_orig_008.png]

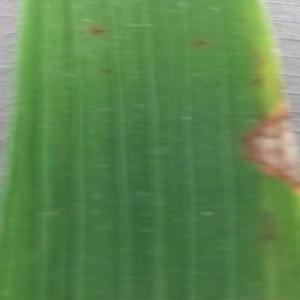

Supplement: S1 Data — (ZIP) [file pone.0295661.s001.zip › rice_images/blast/images/blast_orig_009.jpg]

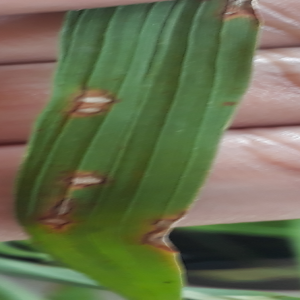

Supplement: S1 Data — (ZIP) [file pone.0295661.s001.zip › rice_images/blast/images/blast_orig_010.png]

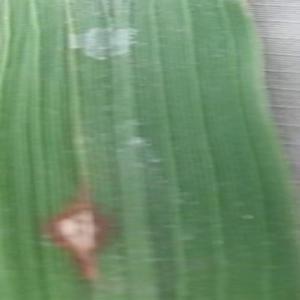

Supplement: S1 Data — (ZIP) [file pone.0295661.s001.zip › rice_images/blast/images/blast_orig_011.jpg]

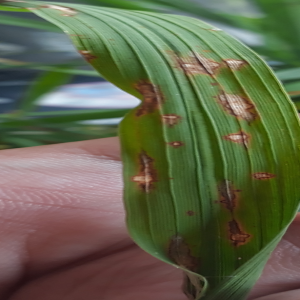

Supplement: S1 Data — (ZIP) [file pone.0295661.s001.zip › rice_images/blast/images/blast_orig_012.png]

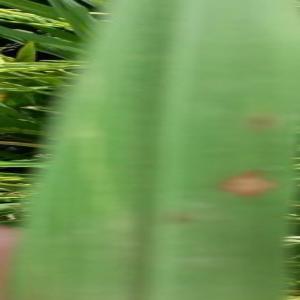

Supplement: S1 Data — (ZIP) [file pone.0295661.s001.zip › rice_images/blast/images/blast_orig_013.jpg]

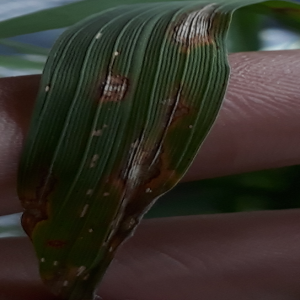

Supplement: S1 Data — (ZIP) [file pone.0295661.s001.zip › rice_images/blast/images/blast_orig_014.png]

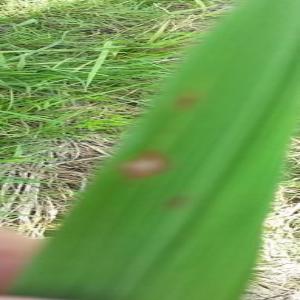

Supplement: S1 Data — (ZIP) [file pone.0295661.s001.zip › rice_images/blast/images/blast_orig_015.jpg]

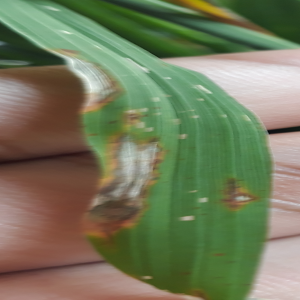

Supplement: S1 Data — (ZIP) [file pone.0295661.s001.zip › rice_images/blast/images/blast_orig_016.png]

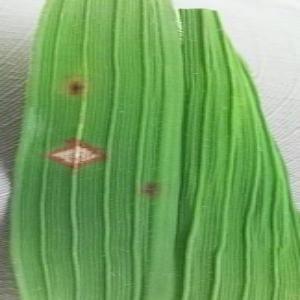

Supplement: S1 Data — (ZIP) [file pone.0295661.s001.zip › rice_images/blast/images/blast_orig_017.jpg]

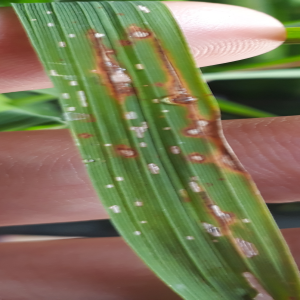

Supplement: S1 Data — (ZIP) [file pone.0295661.s001.zip › rice_images/blast/images/blast_orig_018.png]

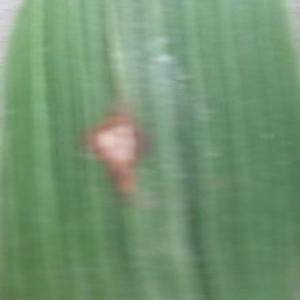

Supplement: S1 Data — (ZIP) [file pone.0295661.s001.zip › rice_images/blast/images/blast_orig_019.jpg]

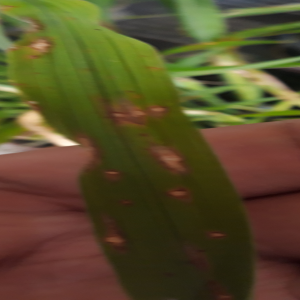

Supplement: S1 Data — (ZIP) [file pone.0295661.s001.zip › rice_images/blast/images/blast_orig_020.png]

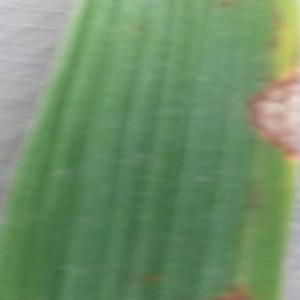

Supplement: S1 Data — (ZIP) [file pone.0295661.s001.zip › rice_images/blast/images/blast_orig_021.jpg]

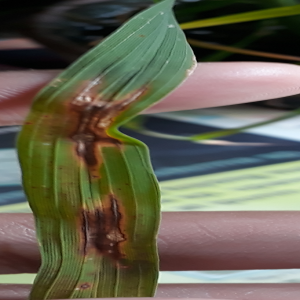

Supplement: S1 Data — (ZIP) [file pone.0295661.s001.zip › rice_images/blast/images/blast_orig_022.png]

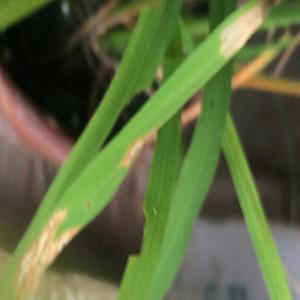

Supplement: S1 Data — (ZIP) [file pone.0295661.s001.zip › rice_images/blast/images/blast_orig_023.jpg]

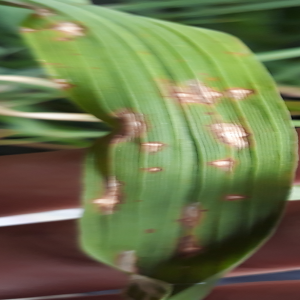

Supplement: S1 Data — (ZIP) [file pone.0295661.s001.zip › rice_images/blast/images/blast_orig_024.png]

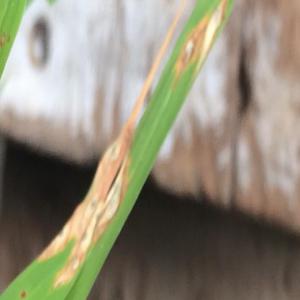

Supplement: S1 Data — (ZIP) [file pone.0295661.s001.zip › rice_images/blast/images/blast_orig_025.jpg]

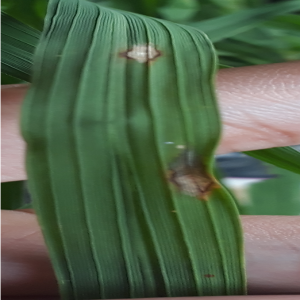

Supplement: S1 Data — (ZIP) [file pone.0295661.s001.zip › rice_images/blast/images/blast_orig_026.png]

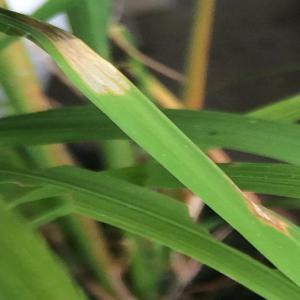

Supplement: S1 Data — (ZIP) [file pone.0295661.s001.zip › rice_images/blast/images/blast_orig_027.jpg]

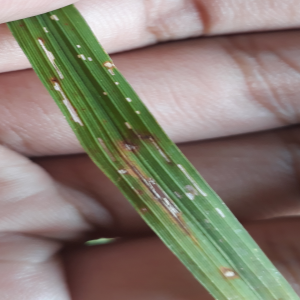

Supplement: S1 Data — (ZIP) [file pone.0295661.s001.zip › rice_images/blast/images/blast_orig_028.png]

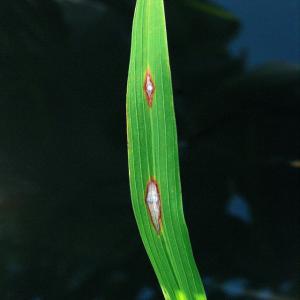

Supplement: S1 Data — (ZIP) [file pone.0295661.s001.zip › rice_images/blast/images/blast_orig_029.jpg]

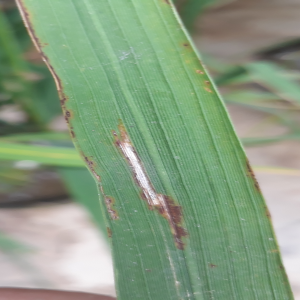

Supplement: S1 Data — (ZIP) [file pone.0295661.s001.zip › rice_images/blast/images/blast_orig_030.png]

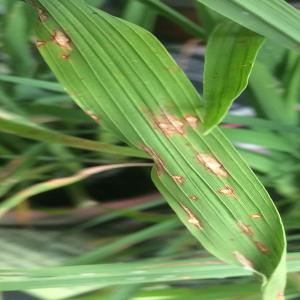

Supplement: S1 Data — (ZIP) [file pone.0295661.s001.zip › rice_images/blast/images/blast_orig_031.JPG]

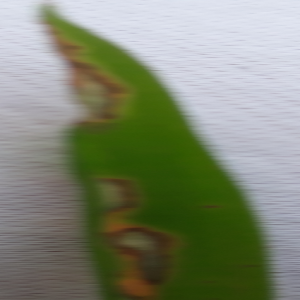

Supplement: S1 Data — (ZIP) [file pone.0295661.s001.zip › rice_images/blast/images/blast_orig_032.png]

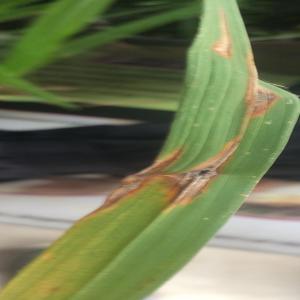

Supplement: S1 Data — (ZIP) [file pone.0295661.s001.zip › rice_images/blast/images/blast_orig_033.JPG]

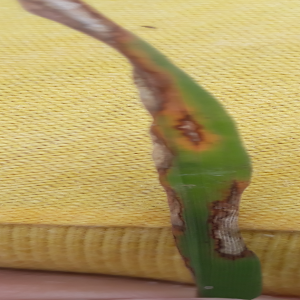

Supplement: S1 Data — (ZIP) [file pone.0295661.s001.zip › rice_images/blast/images/blast_orig_034.png]

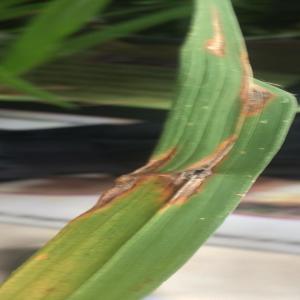

Supplement: S1 Data — (ZIP) [file pone.0295661.s001.zip › rice_images/blast/images/blast_orig_035.JPG]

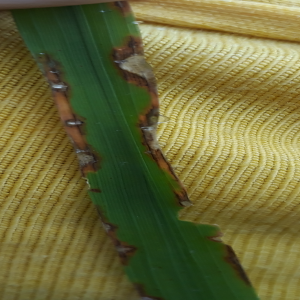

Supplement: S1 Data — (ZIP) [file pone.0295661.s001.zip › rice_images/blast/images/blast_orig_036.png]

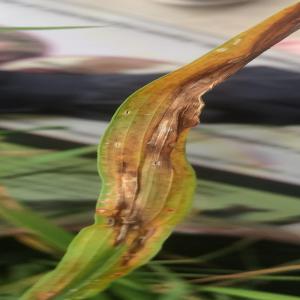

Supplement: S1 Data — (ZIP) [file pone.0295661.s001.zip › rice_images/blast/images/blast_orig_037.JPG]

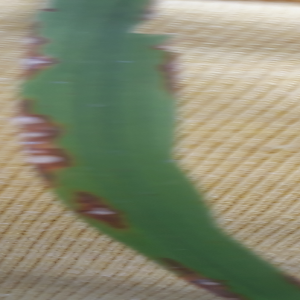

Supplement: S1 Data — (ZIP) [file pone.0295661.s001.zip › rice_images/blast/images/blast_orig_038.png]

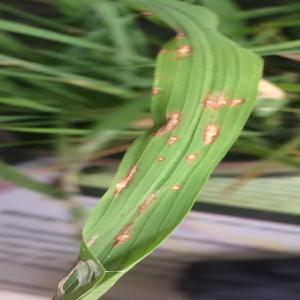

Supplement: S1 Data — (ZIP) [file pone.0295661.s001.zip › rice_images/blast/images/blast_orig_039.JPG]

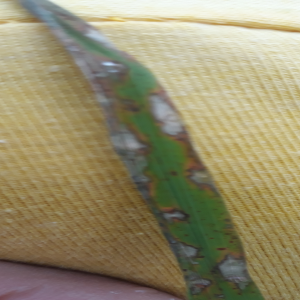

Supplement: S1 Data — (ZIP) [file pone.0295661.s001.zip › rice_images/blast/images/blast_orig_040.png]

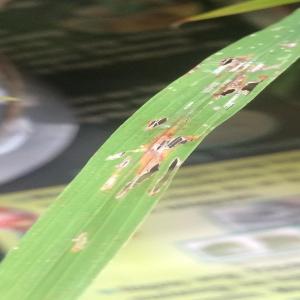

Supplement: S1 Data — (ZIP) [file pone.0295661.s001.zip › rice_images/blast/images/blast_orig_041.JPG]

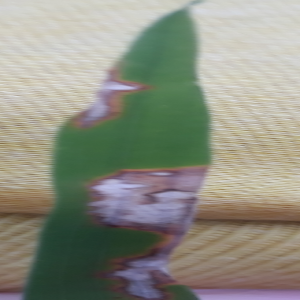

Supplement: S1 Data — (ZIP) [file pone.0295661.s001.zip › rice_images/blast/images/blast_orig_042.png]

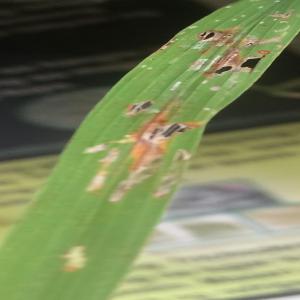

Supplement: S1 Data — (ZIP) [file pone.0295661.s001.zip › rice_images/blast/images/blast_orig_043.JPG]

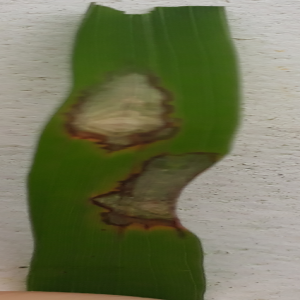

Supplement: S1 Data — (ZIP) [file pone.0295661.s001.zip › rice_images/blast/images/blast_orig_044.png]

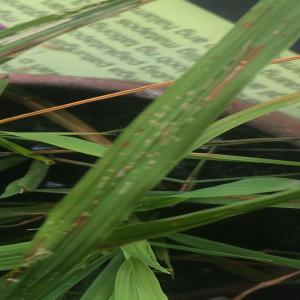

Supplement: S1 Data — (ZIP) [file pone.0295661.s001.zip › rice_images/blast/images/blast_orig_045.JPG]

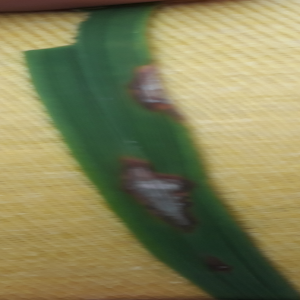

Supplement: S1 Data — (ZIP) [file pone.0295661.s001.zip › rice_images/blast/images/blast_orig_046.png]

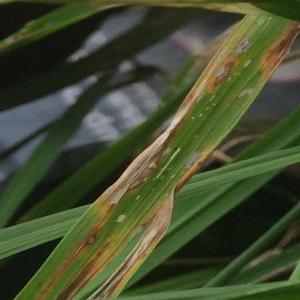

Supplement: S1 Data — (ZIP) [file pone.0295661.s001.zip › rice_images/blast/images/blast_orig_047.jpg]

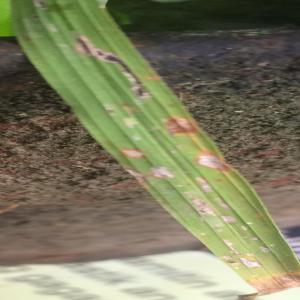

Supplement: S1 Data — (ZIP) [file pone.0295661.s001.zip › rice_images/blast/images/blast_orig_048.JPG]

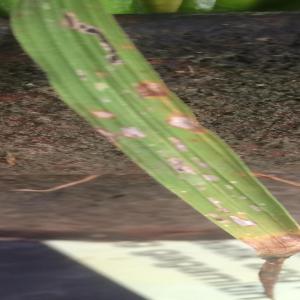

Supplement: S1 Data — (ZIP) [file pone.0295661.s001.zip › rice_images/blast/images/blast_orig_049.JPG]

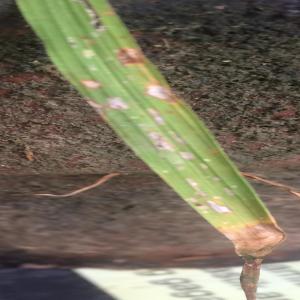

Supplement: S1 Data — (ZIP) [file pone.0295661.s001.zip › rice_images/blast/images/blast_orig_050.JPG]

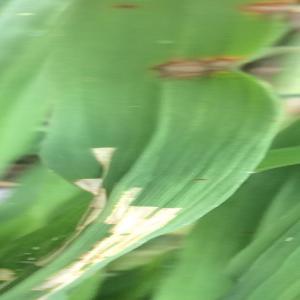

Supplement: S1 Data — (ZIP) [file pone.0295661.s001.zip › rice_images/blast/images/blast_orig_051.JPG]

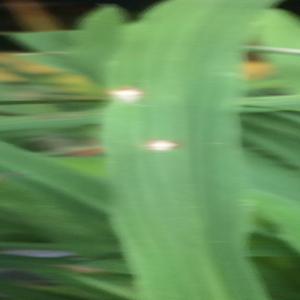

Supplement: S1 Data — (ZIP) [file pone.0295661.s001.zip › rice_images/blast/images/blast_orig_052.jpg]

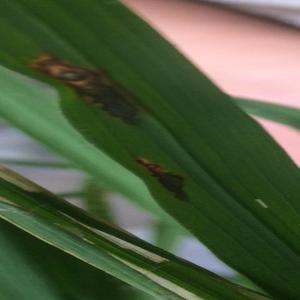

Supplement: S1 Data — (ZIP) [file pone.0295661.s001.zip › rice_images/blast/images/blast_orig_053.JPG]

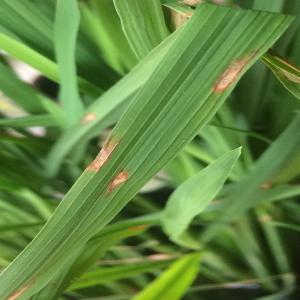

Supplement: S1 Data — (ZIP) [file pone.0295661.s001.zip › rice_images/blast/images/blast_orig_054.JPG]

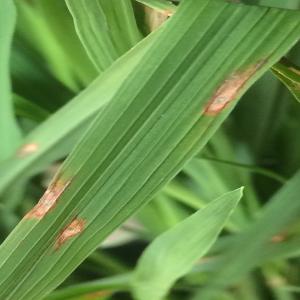

Supplement: S1 Data — (ZIP) [file pone.0295661.s001.zip › rice_images/blast/images/blast_orig_055.jpg]

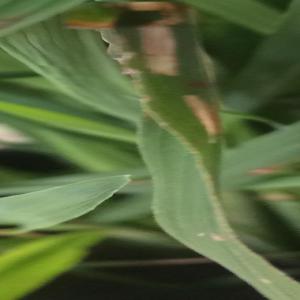

Supplement: S1 Data — (ZIP) [file pone.0295661.s001.zip › rice_images/blast/images/blast_orig_056.JPG]

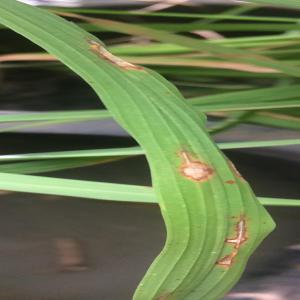

Supplement: S1 Data — (ZIP) [file pone.0295661.s001.zip › rice_images/blast/images/blast_orig_057.JPG]

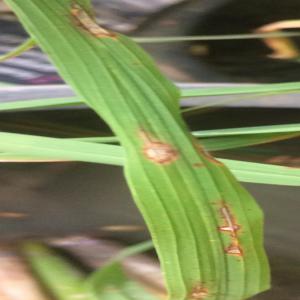

Supplement: S1 Data — (ZIP) [file pone.0295661.s001.zip › rice_images/blast/images/blast_orig_058.JPG]

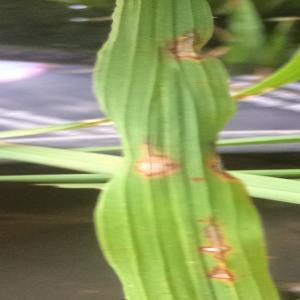

Supplement: S1 Data — (ZIP) [file pone.0295661.s001.zip › rice_images/blast/images/blast_orig_059.JPG]

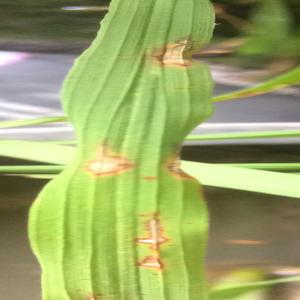

Supplement: S1 Data — (ZIP) [file pone.0295661.s001.zip › rice_images/blast/images/blast_orig_060.JPG]

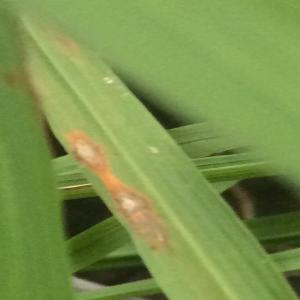

Supplement: S1 Data — (ZIP) [file pone.0295661.s001.zip › rice_images/blast/images/blast_orig_061.JPG]

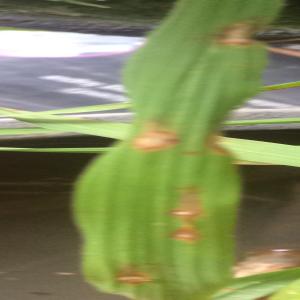

Supplement: S1 Data — (ZIP) [file pone.0295661.s001.zip › rice_images/blast/images/blast_orig_062.jpg]

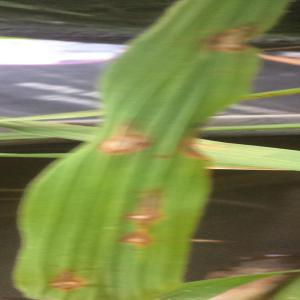

Supplement: S1 Data — (ZIP) [file pone.0295661.s001.zip › rice_images/blast/images/blast_orig_063.JPG]

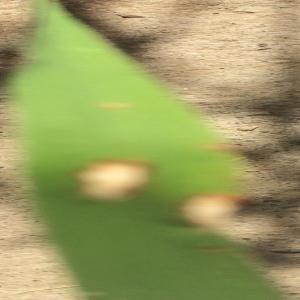

Supplement: S1 Data — (ZIP) [file pone.0295661.s001.zip › rice_images/blast/images/blast_orig_064.JPG]

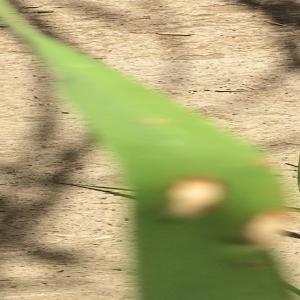

Supplement: S1 Data — (ZIP) [file pone.0295661.s001.zip › rice_images/blast/images/blast_orig_065.JPG]

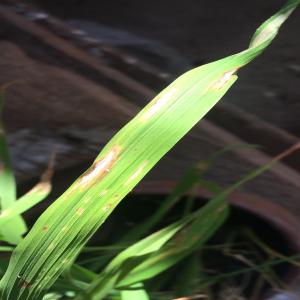

Supplement: S1 Data — (ZIP) [file pone.0295661.s001.zip › rice_images/blast/images/blast_orig_066.JPG]

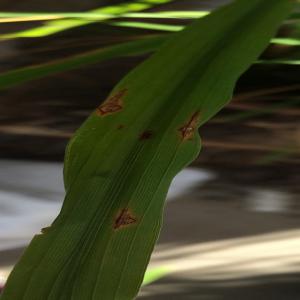

Supplement: S1 Data — (ZIP) [file pone.0295661.s001.zip › rice_images/blast/images/blast_orig_067.JPG]

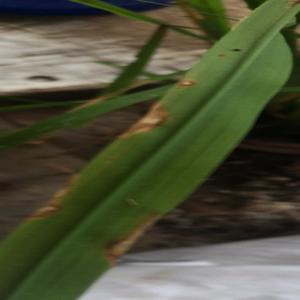

Supplement: S1 Data — (ZIP) [file pone.0295661.s001.zip › rice_images/blast/images/blast_orig_068.JPG]

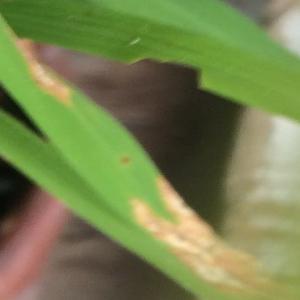

Supplement: S1 Data — (ZIP) [file pone.0295661.s001.zip › rice_images/blast/images/blast_orig_069.JPG]

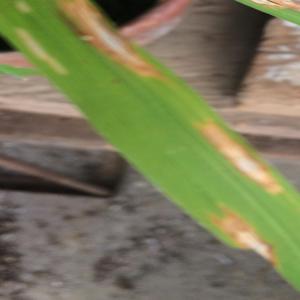

Supplement: S1 Data — (ZIP) [file pone.0295661.s001.zip › rice_images/blast/images/blast_orig_070.JPG]

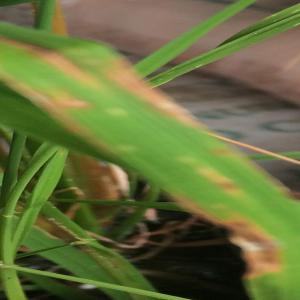

Supplement: S1 Data — (ZIP) [file pone.0295661.s001.zip › rice_images/blast/images/blast_orig_071.JPG]

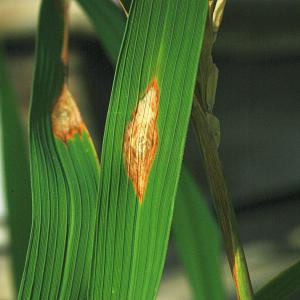

Supplement: S1 Data — (ZIP) [file pone.0295661.s001.zip › rice_images/blast/images/blast_orig_072.jpg]

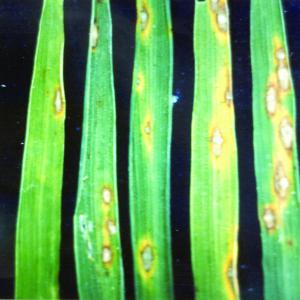

Supplement: S1 Data — (ZIP) [file pone.0295661.s001.zip › rice_images/blast/images/blast_orig_073.jpg]

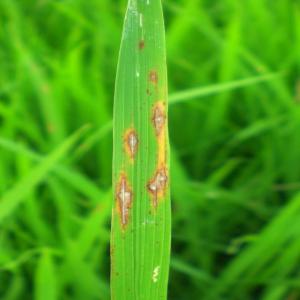

Supplement: S1 Data — (ZIP) [file pone.0295661.s001.zip › rice_images/blast/images/blast_orig_074.jpg]

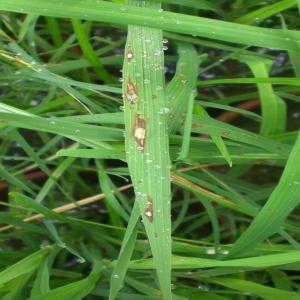

Supplement: S1 Data — (ZIP) [file pone.0295661.s001.zip › rice_images/blast/images/blast_orig_075.jpg]

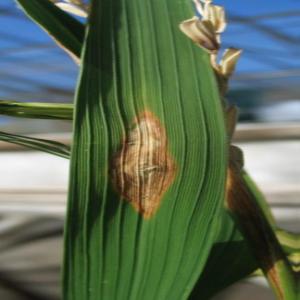

Supplement: S1 Data — (ZIP) [file pone.0295661.s001.zip › rice_images/blast/images/blast_orig_076.jpg]

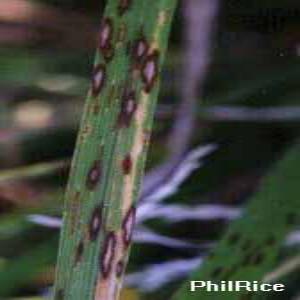

Supplement: S1 Data — (ZIP) [file pone.0295661.s001.zip › rice_images/blast/images/blast_orig_077.jpg]

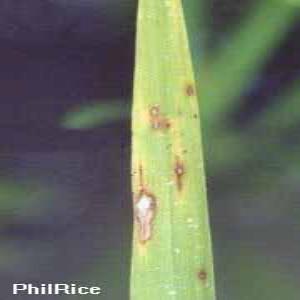

Supplement: S1 Data — (ZIP) [file pone.0295661.s001.zip › rice_images/blast/images/blast_orig_078.jpg]

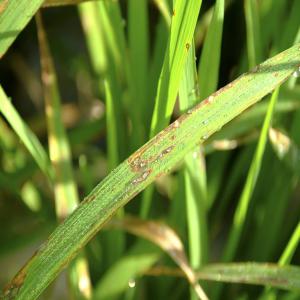

Supplement: S1 Data — (ZIP) [file pone.0295661.s001.zip › rice_images/blast/images/blast_orig_079.jpg]

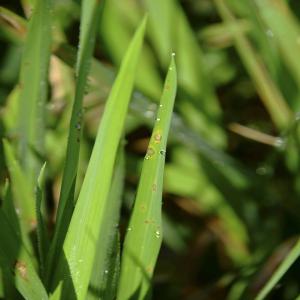

Supplement: S1 Data — (ZIP) [file pone.0295661.s001.zip › rice_images/blast/images/blast_orig_080.jpg]

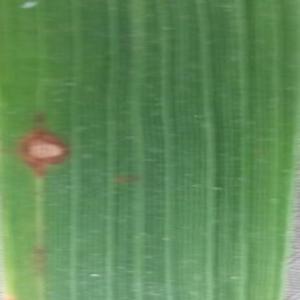

Supplement: S1 Data — (ZIP) [file pone.0295661.s001.zip › rice_images/blast/images/blast_rotated_001.jpg]

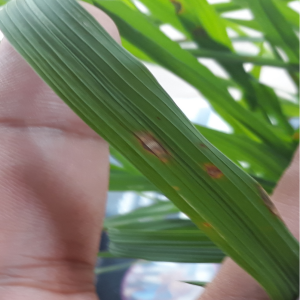

Supplement: S1 Data — (ZIP) [file pone.0295661.s001.zip › rice_images/blast/images/blast_rotated_002.png]

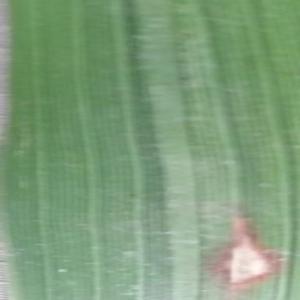

Supplement: S1 Data — (ZIP) [file pone.0295661.s001.zip › rice_images/blast/images/blast_rotated_003.jpg]

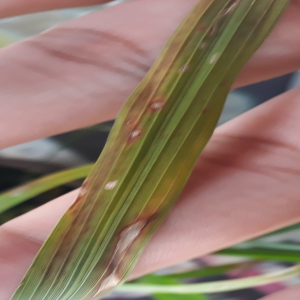

Supplement: S1 Data — (ZIP) [file pone.0295661.s001.zip › rice_images/blast/images/blast_rotated_004.png]

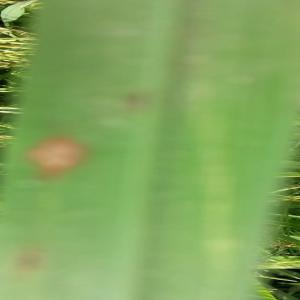

Supplement: S1 Data — (ZIP) [file pone.0295661.s001.zip › rice_images/blast/images/blast_rotated_005.jpg]

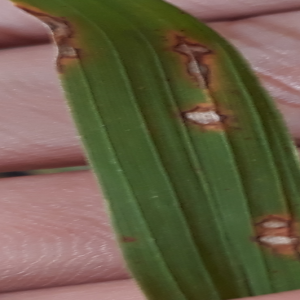

Supplement: S1 Data — (ZIP) [file pone.0295661.s001.zip › rice_images/blast/images/blast_rotated_006.png]

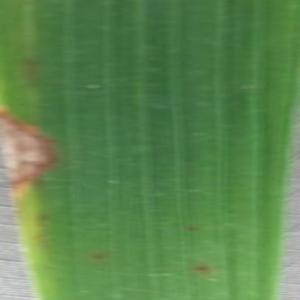

Supplement: S1 Data — (ZIP) [file pone.0295661.s001.zip › rice_images/blast/images/blast_rotated_007.jpg]

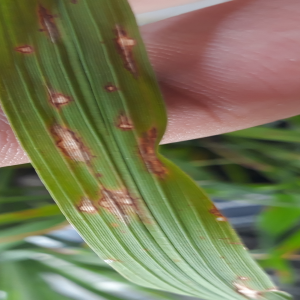

Supplement: S1 Data — (ZIP) [file pone.0295661.s001.zip › rice_images/blast/images/blast_rotated_008.png]

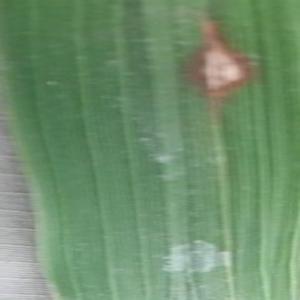

Supplement: S1 Data — (ZIP) [file pone.0295661.s001.zip › rice_images/blast/images/blast_rotated_009.jpg]

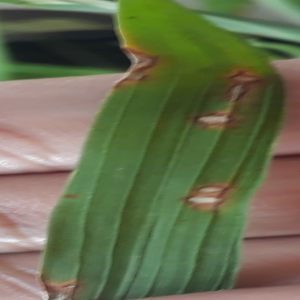

Supplement: S1 Data — (ZIP) [file pone.0295661.s001.zip › rice_images/blast/images/blast_rotated_010.png]

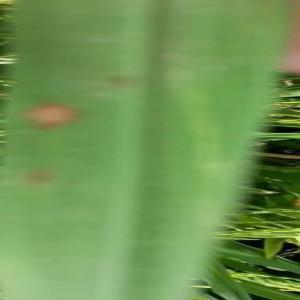

Supplement: S1 Data — (ZIP) [file pone.0295661.s001.zip › rice_images/blast/images/blast_rotated_011.jpg]

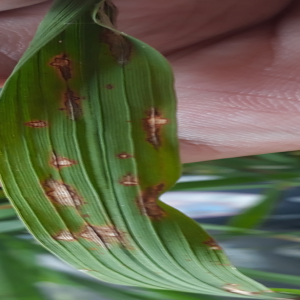

Supplement: S1 Data — (ZIP) [file pone.0295661.s001.zip › rice_images/blast/images/blast_rotated_012.png]

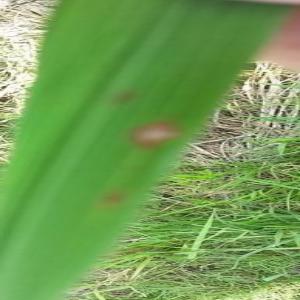

Supplement: S1 Data — (ZIP) [file pone.0295661.s001.zip › rice_images/blast/images/blast_rotated_013.jpg]

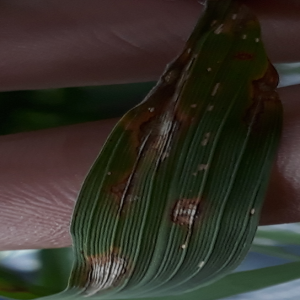

Supplement: S1 Data — (ZIP) [file pone.0295661.s001.zip › rice_images/blast/images/blast_rotated_014.png]

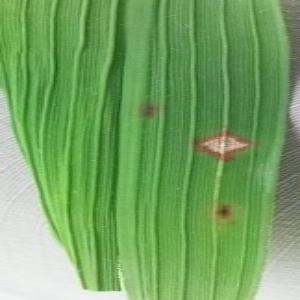

Supplement: S1 Data — (ZIP) [file pone.0295661.s001.zip › rice_images/blast/images/blast_rotated_015.jpg]

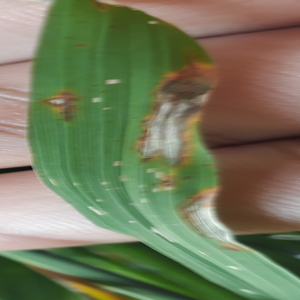

Supplement: S1 Data — (ZIP) [file pone.0295661.s001.zip › rice_images/blast/images/blast_rotated_016.png]

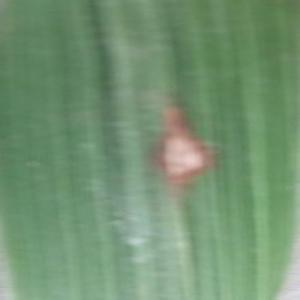

Supplement: S1 Data — (ZIP) [file pone.0295661.s001.zip › rice_images/blast/images/blast_rotated_017.jpg]

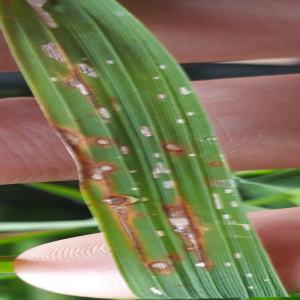

Supplement: S1 Data — (ZIP) [file pone.0295661.s001.zip › rice_images/blast/images/blast_rotated_018.png]

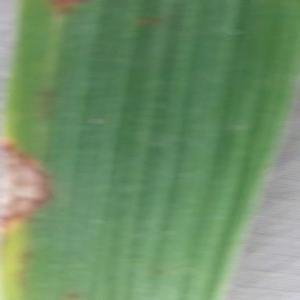

Supplement: S1 Data — (ZIP) [file pone.0295661.s001.zip › rice_images/blast/images/blast_rotated_019.jpg]

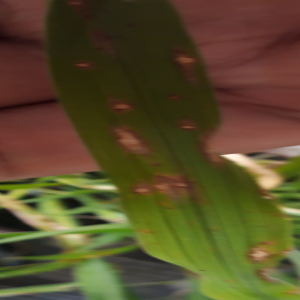

Supplement: S1 Data — (ZIP) [file pone.0295661.s001.zip › rice_images/blast/images/blast_rotated_020.png]
