# Supplementary material for: Research on rice disease recognition based on improved SPPFCSPC-G YOLOv5 network
Source: PLoS One. 2023 Dec 15;18(12):e0295661. doi: 10.1371/journal.pone.0295661 (PMC10723668; doi:10.1371/journal.pone.0295661)
Supplement: Attachment — Submitted filename: Review Comments.pdf [file pone.0295661.s002.pdf]

Review Comments

In this paper, an improved SPP-x YOLOv5 model is proposed for rice disease leaf recognition and detection. By improving the original SPP module and building a new YOLOv5 structure through the Adam optimizer. The proposed new method can improve the accuracy and efficiency of rice virus detection. The article is clear and the content is basically complete, but there are still some problems that need to be improved

Question 1： The innovations proposed in this paper are not convincing enough. The proposed SPP-x structure changes the scale of the MaxPool layer. The original SPP module is 5×5, 9×9 and 13×13 Maxpool. The article modifies it into three identical 5×5 Maxpool. This does not conform to the improvement of the structure, and is more similar to the steps of tuning parameters in essence. Adam has been implemented as an optimizer for neural network training in most of the work, and it cannot be regarded as the innovation of this paper. Based on the structure of YOLOv5, the author should conduct a new refinement of the proposed innovations.

Question 2： This paper improves the detection of rice leaf disease based on YOLOv5. The existing object detection methods are all based on the improvement of YOLOv5, such as [1-2]. Therefore, it is recommended that the author introduce the related YOLOv5-based target detection method in the introduction and compare it with the method proposed in this paper, so as to highlight the advantages of the method in this paper.

[1] Wang, J., Chen, Y., Dong, Z. et al. Improved YOLOv5 network for real-time multi-scale traffic sign detection. Neural Comput & Applic 35, 7853–7865 (2023). https://doi.org/10.1007/s00521-022-08077-5.

[2] Zhu X, Lyu S, Wang X, et al. TPH-YOLOv5: Improved YOLOv5 based on transformer prediction head for object detection on drone-captured scenarios[C]. Proceedings of the IEEE/CVF international conference on computer vision, 2778-2788 (2021). https://doi.org/10.1109/ICCVW54120.2021.00312.

Question 3： The experimental part needs to be richer. It is necessary to compare the method proposed in this paper with the existing detection methods, both qualitatively and quantitatively to illustrate the effectiveness of the proposed method.

Question 4： Some figures in the experimental part are blurred, such as Fig. 4. It is recommended to increase the resolution of the image.
