# Supplementary material for: Research on rice disease recognition based on improved SPPFCSPC-G YOLOv5 network
Source: PLoS One. 2023 Dec 15;18(12):e0295661. doi: 10.1371/journal.pone.0295661 (PMC10723668; doi:10.1371/journal.pone.0295661)
Supplement: Attachment — Submitted filename: Reviewer Attachments.pdf [file pone.0295661.s003.pdf]

1. The change speed of YOLO series models is fast. Now YOLOv8 has been launched. Please elaborate why YOLOv5 is chosen as the main object detection model in this paper
2. The innovation of the improved model in Section 2.3.1 is not enough. The core content is only replacing the original SPP module of YOLOv5
3. The experimental analysis part lacks the performance comparison with other classical single-stage and two-stage models, and the workload is insufficient
4. The final recognition results show the recognition effect of the improved network model, but the number of recognition renderings is insufficient, which can not well reflect the performance improvement of the improved model
