# Supplementary material for: Research on rice disease recognition based on improved SPPFCSPC-G YOLOv5 network
Source: PLoS One. 2023 Dec 15;18(12):e0295661. doi: 10.1371/journal.pone.0295661 (PMC10723668; doi:10.1371/journal.pone.0295661)
Supplement: Attachment — Submitted filename: Response to Reviewers.docx [file pone.0295661.s004.docx]

Dear esteemed journal editor and reviewers

Firstly, we would like to sincerely thank you for reviewing our paper and providing valuable feedback. Your professional opinions and suggestions have been of great help to our research.

In our response, we have addressed each question raised by the reviewers and made necessary revisions and improvements based on your suggestions. The following is the work we have done in response to each question:

**Reviewer #1:**

1.The innovations proposed in this paper are not convincing enough. The proposed SPP-x structure changes the scale of the MaxPool layer. The original SPP module is 5×5, 9×9 and 13×13 Maxpool. The article modifies it into three identical 5×5 Maxpool. This does not conform to the improvement of the structure, and is more similar to the steps of tuning parameters in essence. Adam has been implemented as an optimizer for neural network training in most of the work, and it cannot be regarded as the innovation of this paper. Based on the structure of YOLOv5, the author should conduct a new refinement of the proposed innovations.

**Answer to Question 1:**Thank you for your insightful feedback. We have carefully considered your comments and made significant innovations to the SPP module based on your suggestions. Additionally, we conducted additional experiments to demonstrate the superiority of our improved SPPFCSPC-G module. Through extensive experimentation involving 13 different models, we have validated the effectiveness of our proposed approach.

2.This paper improves the detection of rice leaf disease based on YOLOv5. The existing object detection methods are all based on the improvement of YOLOv5, such as [1-2]. Therefore, it is recommended that the author introduce the related YOLOv5-based target detection method in the introduction and compare it with the method proposed in this paper, so as to highlight the advantages of the method in this paper.[1] Wang, J., Chen, Y., Dong, Z. et al. Improved YOLOv5 network for real-time multi-scale traffic sign detection. Neural Comput & Applic 35, 7853–7865 (2023). <https://doi.org/10.1007/s00521-022-08077-5>.[2] Zhu X, Lyu S, Wang X, et al. TPH-YOLOv5: Improved YOLOv5 based on transformer prediction head for object detection on drone-captured scenarios[C]. Proceedings of the IEEE/CVF international conference on computer vision, 2778-2788 (2021). <https://doi.org/10.1109/ICCVW54120.2021.00312>.

**Answer to Question 2:**Thank you very much for your feedback. We have added an introduction to the latest developments in the SPP module in the [Introduction] section of the paper, including the evolution, structural ideas, and improvements of various versions of SPP. Additionally, in the [Improved SPP structure] section, we have provided a comprehensive explanation of our newly developed SPPFCSPC-G module.

Once again, we sincerely appreciate your valuable suggestions, as they have played a crucial role in improving our paper. We will continue to work diligently and incorporate your guidance to further enhance the content of the manuscript.

3.The experimental part needs to be richer. It is necessary to compare the method proposed in this paper with the existing detection methods, both qualitatively and quantitatively to illustrate the effectiveness of the proposed method.

**Answer to Question 3:**Thank you for your feedback. Based on your suggestions, we have made significant additions to the experimental section. We have included data from 13 different models, including VGG-16, Resnet-34, and various SPP modules. In this comparison, we have also incorporated SPP modules from YOLOv6 and YOLOv7 into YOLOv5 for experimentation. Ultimately, we have demonstrated the superior performance of the new SPPFCSPC-G module.

We have conducted both quantitative and qualitative comparisons to showcase the effectiveness of the proposed method in this paper. Our experimental results clearly indicate that the SPPFCSPC-G module outperforms other methods and effectively enhances the accuracy and efficiency of detection.

Once again, we express our gratitude for your valuable feedback. Your suggestions have played a pivotal role in improving the quality and content of our research.

4.Some figures in the experimental part are blurred, such as Fig. 4. It is recommended to increase the resolution of the image.

**Answer to Question 4:**Thank you for your feedback. We have re-created the figures in the experimental section and increased the resolution of the images. You will see these updated figures in the revised version of the paper.

Once again, we express our gratitude for your valuable suggestions. Your feedback has played a crucial role in improving the quality and presentation of our paper.

**Reviewer #2:**

1.The change speed of YOLO series models is fast. Now YOLOv8 has been launched. Please elaborate why YOLOv5 is chosen as the main object detection model in this paper

**Answer to Question 1:**Thank you for your suggestion. (1) YOLOv5 has been widely used and studied in various real-world scenarios since its release in 2020. It has been widely accepted and researched by scholars worldwide. Researchers who study YOLO are familiar with its network structure and various improvements. Thus, YOLOv5 remains of great research value.

(2) The network structure of YOLOv5 (BackBone, Neck, Head) is still considered a classic. Subsequent versions like YOLOv6/v7/v8 are based on the foundation of YOLOv5 with upgrades, such as adding attention mechanisms, improving loss functions, and enhancing modules like SPP, Conv, and CBL in the backbone. Decoupling mechanisms are also employed.

(3) YOLOv6 source code is mostly derived from YOLOv5, with certain components and structures improved. YOLOv7 maintains a similar overall structure to YOLOv5, using the backbone+FPN+PAN+head×3 format (consistent with YOLOv5). YOLOv8 also builds upon YOLOv5 and introduces new features like pose estimation and semantic segmentation. Therefore, YOLOv5 retains significant practical research value.

Once again, we appreciate your valuable feedback, and we believe YOLOv5's significance in our research remains substantial.

2. The innovation of the improved model in Section 2.3.1 is not enough. The core content is only replacing the original SPP module of YOLOv5

**Answer to Question 2:**Thank you for your feedback. In the [Improved SPP structure] section of the paper, we made significant innovations to the SPP module and introduced the SPPFCSPC-G module. We didn't simply replace the original SPP module in YOLOv5; instead, we conducted in-depth optimizations and improvements. Additionally, we experimented by incorporating the SPP modules from YOLOv6 and YOLOv7 into YOLOv5, and the results confirmed that our new SPPFCSPC-G module outperforms them.

We firmly believe that these improvements have made a significant contribution to enhancing the performance and accuracy of our proposed method in this paper. We appreciate your interest in our research and valuable feedback.

3. The experimental analysis part lacks the performance comparison with other classical single-stage and two-stage models, and the workload is insufficient

**Answer to Question 3:**Thank you for your feedback. We have included data from 13 different models, including VGG-16, Resnet-34, and various SPP modules. We also conducted comparative experiments with the SPP modules from YOLOv6 and YOLOv7, and tested them by integrating into YOLOv5. The results showed that our new SPPFCSPC-G module outperforms the others.

Once again, we sincerely appreciate your valuable feedback, and we have made the necessary revisions in the revised version of the paper.

4. The final recognition results show the recognition effect of the improved network model, but the number of recognition renderings is insufficient, which can not well reflect the performance improvement of the improved model

**Answer to Question 4:**Thank you for your valuable feedback.(1) Based on your suggestion, we have included more model performance parameters in the revised paper, which are presented in Table 1, Table 2, Table 3, and Table 4. Through these data, we have thoroughly discussed the advancements of our model, covering various aspects such as Precision, Recall, Loss, mAP, GPU-mem, Parameters, GFLOPs, Params., training speed, and inference speed. Specific data and results can be found in the new version of our submitted paper.

(2) In response to your recommendation, we have added more recognition result images in the paper, as shown in Figure 7. Through these additional recognition result images, we aim to better demonstrate the performance improvement of our improved model.

Once again, we appreciate your attention to our paper and your valuable feedback. Your suggestions have played a crucial role in enhancing the quality and presentation of our research. We have made the necessary modifications in the paper accordingly.

**Reviewer #3:**

1.Authors should improve the organization of the Paper and the writing style.

**Answer to Question 1:**Thank you for your suggestion. We have made improvements to the writing style of the paper, aiming to make the new version more clear and readable. Once again, we appreciate your attention and valuable feedback on our paper.

2. The Introduction should cover problem definition, motivation and contributions.

**Answer to Question 2:**Thank you for your feedback. We have revised the Introduction section to include problem definition, motivation, and contributions. These additions aim to provide a more comprehensive and informative introduction to our research. Once again, we appreciate your valuable input on our paper.

3. The authors should add a seperate section for Related work and cover all the latest and relevant works such SE_SPNet and ktehr studies.

**Answer to Question 3:**Thank you for your feedback. We have added a new section in the Introduction, providing an overview and review of the latest developments in the SPP module. This includes the ideas and improvements proposed by scholars for various versions of the SPP structure. We hope these additions will better demonstrate the position and significance of our research in the context of existing work. Once again, we appreciate your attention to our paper and your valuable feedback.

4. Add a comparative table in the Retaled work section. Highlighting limitations of the existing works.

**Answer to Question 4:**Thank you for your feedback. We have included several models in our study, and in the revised version of the paper, we have provided comparative tables, specifically Table 1, Table 2, Table 3, and Table 4. These tables demonstrate the advanced performance of our model in various aspects, including Precision, Recall, Loss, mAP, GPU-mem, Parameters, GFLOPs, Params., training speed, and inference speed. For specific data, please refer to our newly submitted paper.

5. Please provide the rationale behind selection of the proposed model.

**Answer to Question 5:**Thank you for your feedback. In the revised version of the paper, we have provided a detailed description of the improvements made to the SPP module in the [Improved SPP structure] section. We have also included Figure 3, Figure 4, and Equation (1) in the revised paper to illustrate the rationale behind the selection of our proposed model. These additions aim to provide a clear and comprehensive explanation of why we chose this particular model for our research.

6. Discuss computational complexity of the proposed work and compare it with existing works.

**Answer to Question 6:**Thank you for your suggestion. In the newly submitted paper, you can find experimental data related to computational complexity parameters such as GPU-mem, Parameters, GFLOPs, and Params. in Table 3 and Table 4. We have also provided corresponding explanations to compare the computational complexity of our proposed work with existing works.

7. Compare your work with state of the art techniques and other similar works.

**Answer to Question 7:**Thank you for your feedback. We have included several models and provided comparative performance data in Table 1, which demonstrates the advanced performance of our model in terms of Precision, Recall, Loss, mAP, GPU-mem, Parameters, GFLOPs, Params., training speed, and inference speed. Please refer to the revised paper for specific data and detailed discussions. Once again, we appreciate your attention to our paper and your valuable feedback.

8. Discuss limitations of the proposed technique.

**Answer to Question 8:**Thank you for your feedback. In the [Conclusion] section of the paper, we have discussed the limitations of our technique. We address the issue of decreased inference speed caused by the new model and the significant performance variation of the optimizer on the dataset. Our research team will further explore and investigate these factors in future work.
