# Supplementary material for: Research on rice disease recognition based on improved SPPFCSPC-G YOLOv5 network
Source: PLoS One. 2023 Dec 15;18(12):e0295661. doi: 10.1371/journal.pone.0295661 (PMC10723668; doi:10.1371/journal.pone.0295661)
Supplement: Attachment — Submitted filename: Response to Reviewers.docx [file pone.0295661.s005.docx]

Dear esteemed journal editor and reviewers

Firstly, we would like to sincerely thank you for reviewing our paper and providing valuable feedback. Your professional opinions and suggestions have been of great help to our research.

In our response, we have addressed each question raised by the reviewers and made necessary revisions and improvements based on your suggestions. The following is the work we have done in response to each question:

**Reviewer #4:**

**Question 1:** First of all, to demonstrate the effectiveness of proposed approach authors must compare with previous research work(s) on same dataset, say previous few architectures/improved architectures on one/more same dataset (in this work, rice-disease dataset). Authors should demonstrate the improvement against similar works.

**Answer to Question 1:**Thank you for your valuable feedback. Our research work is carried out in conjunction with our fund project. Our research direction is mainly to use computer vision technology to identify and detect the disease of rice in specific scenarios.

In the popular COCO, ImageNet, and PASCAL datasets, the objects in each image are not rice (our experimental research object is rice). Although there are some rice objects in the COCO dataset, there are also other objects in each image, and their quantity is huge. Although such public datasets are good for verifying the generalization ability of models, our work can only perform image recognition on a specific rice dataset. Therefore, by training a dedicated rice dataset, we can obtain model parameters and prediction models that are more in line with actual conditions, thereby improving the targeting and accuracy of the model.

**Question 2：**What is the motivation against selecting VGG-16 and/or ResNet-34, why not other VGG-x/ResNet-x architectures ?[2-1] Even, baseline Yolov-x recent architectures also have been modified. Please demonstrate a comparison against Recent YOLOv-x architectures as well like, utilizing SAHI , Yolov8, Yolo-NAS or ViT based models. Authors should not use an arbitrary choice of models to demonstrate improvement. [2-2]Also, if improvement metric should be considered with previous modules , those are not convincing at all (as authors only proposed improvement on a particular dataset or use case, whereas baseline models generally benchmarked on an universal dataset like PASCAL/COCO etc.). [2-3]The improvement factors to be noticed as .50% against SimSPPF[17] or 1.0% against SPPFCSPC only for precision, 1.1% or 1.3% for recall for same models, +1.2% against SPPCSPC[18] for mAP @0.5 and 0.8% for map @0.95..There is surely no clear trend of improvement to be followed as previous baseline models are also constantly evolving and showing improvement against each other. Therefore, how significant is authors proposed method only on a particular dataset? As authors are mainly claiming improvement of proposed module, please demonstrate the generalizability on a very different public dataset (may be a different domain if possible, like occluded object detection/satellite imagery object detection etc. )[2-4]

**Answer to Question 2:**Thank you for your valuable feedback. Regarding Question 2, we will answer it in four parts.

**Answer to Question 2-1:** In Table 1, we replaced three additional experiments. At the same time, we replaced the original SPPF module with the SPPCSPC-G module designed in this experiment.

Our research work is mainly focused on lightweight models and practical applications, and the trained models will be applied to mobile devices.

Although deeper convolutional neural network structures such as VGG-16/19/30, ResNet-50/101/152, RepVGG-A0/A1/B0/B1, ViTAE-T/S/v2-S represent stronger expression ability and more complex feature learning ability, they may also provide higher computational costs and more difficult-to-train models, with low computational efficiency. The computational resource consumption of deeper network architectures is very large, and the model training time is also very long. At the same time, because of the deep architecture, the models trained are often very large. If a target detection model is deployed on a mobile device, a model that is too large may cause some problems. For example: (1) The storage space of mobile devices is limited, and too large models may occupy too much storage space, thereby affecting the download and use experience of applications. (2) The computing power of mobile devices is relatively limited, and too much computing power may affect the response speed and battery life of applications. The computational cost of target detection models is usually large. If deployed on a mobile device, it may put great pressure on the performance of mobile devices.

In addition, our research work uses computer vision technology to identify and detect the disease of rice in specific scenarios. We do not develop a large network architecture to adapt to different scenarios or different complexity tasks (fine-grained classification, small object detection, multi-object detection, etc.). Our project requirements do not need deeper models. When choosing a suitable model architecture, the project team needs to weigh according to specific tasks and computing resources, without blindly pursuing deep architectures. Therefore we did not choose other VGG-x/ResNet-x architectures.

**Answer to Question 2-2:** (1) First of all, the performance difference between Yolov5 and YOLOv8/v7/v6-3.0 on the COCO dataset is not very significant, as shown in the following figure.

(2) Second, our research work mainly improved one of the SPP modules. Therefore, we used the same type of SPP module as the comparison module (which also includes the SPP module in the latest YOLOv7), and then conducted experiments and verification under the YOLOv5 framework. The final training results are shown in the following table. Precision, recall, and mAP values are very high on our dataset, so the project team considers using YOLOv8/v7 to verify our SPPCSPC-G module on the rice dataset, and it is difficult to further improve performance.

| **Framework** | **Model** | Optimizer | *SteadyEpoch* | *Precision* | *Recall* | $mAP_{0.5}^{val}$ | $mAP_{0.95}^{val}$ |
| --- | --- | --- | --- | --- | --- | --- | --- |
| YOLOv5-s | SPPFCSPC-G(Our) | SGD | 200 | 96.20*%* | 99.2*%* | 98.60*%* | 86.80*%* |

**Answer to Question 2-3:** Our research work is based on our experimental fund projects (1) (2) (3). Our research direction is mainly to use computer vision technology to identify and detect the disease of rice in specific scenarios.

Therefore, the project team established a rice disease dataset to solve the problem of insufficient traditional data. Although traditional generalized datasets (COCO/Imagenet) contain a large amount of data, they cannot provide sufficient data on rice growth and breeding. Therefore, by training a dedicated rice dataset, the problem of insufficient data in the rice field can be solved, and the training effect and prediction accuracy of the model can be improved. Since the growth environment and growth situation of rice are relatively complex, more refined data is needed to support model training and prediction. Training a dedicated rice dataset can provide more refined data, including various details and features during rice growth, thereby better supporting model training and prediction.

(1) Research project on intelligent monitoring and early warning system for rice diseases and insect pests based on meteorological conditions

(2) Intelligent agricultural trusted tracking system based on blockchain

(3) Key technology research on corn seed selection based on convolutional neural network

**Answer to Question 2-4:** We will show the models trained on the COCO dataset at different network depths of YOLOv5-s/x/l to identify rice disease leaves. As shown in the figure below, the models trained on the COCO dataset did not accurately identify and detect the research object for rice leaves using YOLOv5-s/x. Although YOLOv5-l recognized the object in the first two figures, almost all of them were incorrect. Therefore, this is why the project team needs to train on a specific dataset.

Original drawing

 YOLOv5-S

 YOLOv5-X

 YOLOv5-L

**All errors of objects**

Although training on public datasets can detect the model’s generalization ability, our project fund’s research objects are only rice and corn. Therefore, the project team did not show the generalization ability of the SPPFCSPC-G module on public datasets in the paper.

**Question 3：**The citations in Table 1is misleading to readers as [16][17][18][20][21] are not directly related to rise-disease detection application and mostly baseline models. Readers may think authors compared with previous related research works (which should be more relevant).

**Answer to Question 3:** Thank you for your valuable feedback. We have removed the unreasonable reference identifiers in Table 1 and revised and improved Table 3 to make it more standardized.

Thank you again for your valuable feedback.
