# Supplementary material for: Research on rice disease recognition based on improved SPPFCSPC-G YOLOv5 network
Source: PLoS One. 2023 Dec 15;18(12):e0295661. doi: 10.1371/journal.pone.0295661 (PMC10723668; doi:10.1371/journal.pone.0295661)
Supplement: Peer review history

# Peer review: Decision Letter 0

**DOI:** 10.1371/journal.pone.0295661.r001

**Authors:** Sunder Ali Khowaja

30 Jun 2023

PONE-D-23-12038Research on Rice Disease Recognition based on Improved SPP-x YOLOv5 NetworkPLOS ONE

Dear Dr. Zhang,

Thank you for submitting your manuscript to PLOS ONE. After careful consideration, we feel that it has merit but does not fully meet PLOS ONE’s publication criteria as it currently stands. Therefore, we invite you to submit a revised version of the manuscript that addresses the points raised during the review process.

==============================

ACADEMIC EDITOR:

The reviewers have completed their reviews. It has been suggested that the manuscript should undergo major revisions in ordered to be considered for second round of review. Therefore, authors are requested to make necessary changes in the manuscript and prepare point-to-point responses with respect to the corresponding concerns raised by the reviewers. . 

==============================

Please submit your revised manuscript by Aug 14 2023 11:59PM. If you will need more time than this to complete your revisions, please reply to this message or contact the journal office at plosone@plos.org. When you're ready to submit your revision, log on to https://www.editorialmanager.com/pone/ and select the 'Submissions Needing Revision' folder to locate your manuscript file.

Please include the following items when submitting your revised manuscript:

- A rebuttal letter that responds to each point raised by the academic editor and reviewer(s). You should upload this letter as a separate file labeled 'Response to Reviewers'.
- A marked-up copy of your manuscript that highlights changes made to the original version. You should upload this as a separate file labeled 'Revised Manuscript with Track Changes'.
- An unmarked version of your revised paper without tracked changes. You should upload this as a separate file labeled 'Manuscript'.

If you would like to make changes to your financial disclosure, please include your updated statement in your cover letter. Guidelines for resubmitting your figure files are available below the reviewer comments at the end of this letter.

If applicable, we recommend that you deposit your laboratory protocols in protocols.io to enhance the reproducibility of your results. Protocols.io assigns your protocol its own identifier (DOI) so that it can be cited independently in the future. For instructions see: https://journals.plos.org/plosone/s/submission-guidelines#loc-laboratory-protocols. Additionally, PLOS ONE offers an option for publishing peer-reviewed Lab Protocol articles, which describe protocols hosted on protocols.io. Read more information on sharing protocols at https://plos.org/protocols?utm_medium=editorial-email&utm_source=authorletters&utm_campaign=protocols.

We look forward to receiving your revised manuscript.

Kind regards,

Sunder Ali Khowaja, Ph.D.

Academic Editor

PLOS ONE

Journal Requirements:

When submitting your revision, we need you to address these additional requirements.

1. Please ensure that your manuscript meets PLOS ONE's style requirements, including those for file naming. The PLOS ONE style templates can be found at

https://journals.plos.org/plosone/s/file?id=wjVg/PLOSOne_formatting_sample_main_body.pdf and

https://journals.plos.org/plosone/s/file?id=ba62/PLOSOne_formatting_sample_title_authors_affiliations.pdf.

2. Thank you for stating in your Funding Statement:

“This work was supported by science and technology development plan project of Science and Technology Department of Jilin Province, project name: Research on intelligent monitoring and early warning system of rice diseases and pests based on meteorological conditions, project No. 20210203211SF. Intelligent agriculture trusted traceability system based on blockchain No.20220202036NC. Science and technology project of Jilin Provincial Department of Education, Project Name: Research on key technology of maize kernel selection based on convolution neural network, Project No. JJKH20210335KJ.

The funders had no role in study design, data collection and analysis, decision to publish, or preparation of the manuscript.”

Please provide an amended statement that declares *all* the funding or sources of support (whether external or internal to your organization) received during this study, as detailed online in our guide for authors at http://journals.plos.org/plosone/s/submit-now.  Please also include the statement “There was no additional external funding received for this study.” in your updated Funding Statement.

Please include your amended Funding Statement within your cover letter. We will change the online submission form on your behalf.

3. Please include captions for your Supporting Information files at the end of your manuscript, and update any in-text citations to match accordingly. Please see our Supporting Information guidelines for more information: http://journals.plos.org/plosone/s/supporting-information.

[Note: HTML markup is below. Please do not edit.]

Reviewers' comments:

Reviewer's Responses to Questions

**Comments to the Author**

1. Is the manuscript technically sound, and do the data support the conclusions?

The manuscript must describe a technically sound piece of scientific research with data that supports the conclusions. Experiments must have been conducted rigorously, with appropriate controls, replication, and sample sizes. The conclusions must be drawn appropriately based on the data presented. 

Reviewer #1: Yes

Reviewer #2: Yes

Reviewer #3: Partly

**********

2. Has the statistical analysis been performed appropriately and rigorously? 

Reviewer #1: Yes

Reviewer #2: Yes

Reviewer #3: No

**********

3. Have the authors made all data underlying the findings in their manuscript fully available?

The PLOS Data policy requires authors to make all data underlying the findings described in their manuscript fully available without restriction, with rare exception (please refer to the Data Availability Statement in the manuscript PDF file). The data should be provided as part of the manuscript or its supporting information, or deposited to a public repository. For example, in addition to summary statistics, the data points behind means, medians and variance measures should be available. If there are restrictions on publicly sharing data—e.g. participant privacy or use of data from a third party—those must be specified.

Reviewer #1: Yes

Reviewer #2: Yes

Reviewer #3: No

**********

4. Is the manuscript presented in an intelligible fashion and written in standard English?

PLOS ONE does not copyedit accepted manuscripts, so the language in submitted articles must be clear, correct, and unambiguous. Any typographical or grammatical errors should be corrected at revision, so please note any specific errors here.

Reviewer #1: Yes

Reviewer #2: Yes

Reviewer #3: No

**********

5. Review Comments to the Author

Please use the space provided to explain your answers to the questions above. You may also include additional comments for the author, including concerns about dual publication, research ethics, or publication ethics. (Please upload your review as an attachment if it exceeds 20,000 characters)

Reviewer #1: In this paper, an improved SPP-x YOLOv5 model is proposed for rice disease leaf recognition and detection. By improving the original SPP module and building a new YOLOv5 structure through the Adam optimizer. The proposed new method can improve the accuracy and efficiency of rice virus detection. The article is clear and the content is basically complete, but there are still some problems that need to be improved

Question 1：The innovations proposed in this paper are not convincing enough. The proposed SPP-x structure changes the scale of the MaxPool layer. The original SPP module is 5×5, 9×9 and 13×13 Maxpool. The article modifies it into three identical 5×5 Maxpool. This does not conform to the improvement of the structure, and is more similar to the steps of tuning parameters in essence. Adam has been implemented as an optimizer for neural network training in most of the work, and it cannot be regarded as the innovation of this paper. Based on the structure of YOLOv5, the author should conduct a new refinement of the proposed innovations.

Question 2：This paper improves the detection of rice leaf disease based on YOLOv5. The existing object detection methods are all based on the improvement of YOLOv5, such as [1-2]. Therefore, it is recommended that the author introduce the related YOLOv5-based target detection method in the introduction and compare it with the method proposed in this paper, so as to highlight the advantages of the method in this paper.

[1] Wang, J., Chen, Y., Dong, Z. et al. Improved YOLOv5 network for real-time multi-scale traffic sign detection. Neural Comput & Applic 35, 7853–7865 (2023). https://doi.org/10.1007/s00521-022-08077-5.

[2] Zhu X, Lyu S, Wang X, et al. TPH-YOLOv5: Improved YOLOv5 based on transformer prediction head for object detection on drone-captured scenarios[C]. Proceedings of the IEEE/CVF international conference on computer vision, 2778-2788 (2021). https://doi.org/10.1109/ICCVW54120.2021.00312.

Question 3：The experimental part needs to be richer. It is necessary to compare the method proposed in this paper with the existing detection methods, both qualitatively and quantitatively to illustrate the effectiveness of the proposed method.

Question 4：Some figures in the experimental part are blurred, such as Fig. 4. It is recommended to increase the resolution of the image.

Reviewer #2: 1. The change speed of YOLO series models is fast. Now YOLOv8 has been launched. Please elaborate why YOLOv5 is chosen as the main object detection model in this paper

2. The innovation of the improved model in Section 2.3.1 is not enough. The core content is only replacing the original SPP module of YOLOv5

3. The experimental analysis part lacks the performance comparison with other classical single-stage and two-stage models, and the workload is insufficient

4. The final recognition results show the recognition effect of the improved network model, but the number of recognition renderings is insufficient, which can not well reflect the performance improvement of the improved model

Reviewer #3: My comments are as follows:

1. Authors should improve the organization of the Paper and the writing style.

2. The Introduction should cover problem definition, motivation and contributions.

3. The authors should add a seperate section for Related work and cover all the latest and relevant works such SE_SPNet and ktehr studies.

4. Add a comparative table in the Retaled work section. Highlighting limitations of the existing works.

5. Please provide the rationale behind selection of the proposed model.

6. Discuss computational complexity of the proposed work and compare it with existing works.

7. Compare your work with state of the art techniques and other similar works.

8. Discuss limitations of the proposed technique.

**********

6. PLOS authors have the option to publish the peer review history of their article (what does this mean?). If published, this will include your full peer review and any attached files.

If you choose “no”, your identity will remain anonymous but your review may still be made public.

**Do you want your identity to be public for this peer review?** For information about this choice, including consent withdrawal, please see our Privacy Policy.

Reviewer #1: No

Reviewer #2: No

Reviewer #3: No

**********

[NOTE: If reviewer comments were submitted as an attachment file, they will be attached to this email and accessible via the submission site. Please log into your account, locate the manuscript record, and check for the action link "View Attachments". If this link does not appear, there are no attachment files.]

While revising your submission, please upload your figure files to the Preflight Analysis and Conversion Engine (PACE) digital diagnostic tool, https://pacev2.apexcovantage.com/. PACE helps ensure that figures meet PLOS requirements. To use PACE, you must first register as a user. Registration is free. Then, login and navigate to the UPLOAD tab, where you will find detailed instructions on how to use the tool. If you encounter any issues or have any questions when using PACE, please email PLOS at figures@plos.org. Please note that Supporting Information files do not need this step.

Attachment Submitted filename: Review Comments.pdf Click here for additional data file.

Attachment Submitted filename: Reviewer Attachments.pdf Click here for additional data file.

# Author comment: Author response to Decision Letter 0

**DOI:** 10.1371/journal.pone.0295661.r002

24 Jul 2023

Dear esteemed journal editor and reviewers

Firstly, we would like to sincerely thank you for reviewing our paper and providing valuable feedback. Your professional opinions and suggestions have been of great help to our research.

In our response, we have addressed each question raised by the reviewers and made necessary revisions and improvements based on your suggestions. The following is the work we have done in response to each question:

Reviewer #1: 

1.The innovations proposed in this paper are not convincing enough. The proposed SPP-x structure changes the scale of the MaxPool layer. The original SPP module is 5×5, 9×9 and 13×13 Maxpool. The article modifies it into three identical 5×5 Maxpool. This does not conform to the improvement of the structure, and is more similar to the steps of tuning parameters in essence. Adam has been implemented as an optimizer for neural network training in most of the work, and it cannot be regarded as the innovation of this paper. Based on the structure of YOLOv5, the author should conduct a new refinement of the proposed innovations.

Answer to Question 1:Thank you for your insightful feedback. We have carefully considered your comments and made significant innovations to the SPP module based on your suggestions. Additionally, we conducted additional experiments to demonstrate the superiority of our improved SPPFCSPC-G module. Through extensive experimentation involving 13 different models, we have validated the effectiveness of our proposed approach.

2.This paper improves the detection of rice leaf disease based on YOLOv5. The existing object detection methods are all based on the improvement of YOLOv5, such as [1-2]. Therefore, it is recommended that the author introduce the related YOLOv5-based target detection method in the introduction and compare it with the method proposed in this paper, so as to highlight the advantages of the method in this paper.[1] Wang, J., Chen, Y., Dong, Z. et al. Improved YOLOv5 network for real-time multi-scale traffic sign detection. Neural Comput & Applic 35, 7853–7865 (2023). https://doi.org/10.1007/s00521-022-08077-5.[2] Zhu X, Lyu S, Wang X, et al. TPH-YOLOv5: Improved YOLOv5 based on transformer prediction head for object detection on drone-captured scenarios[C]. Proceedings of the IEEE/CVF international conference on computer vision, 2778-2788 (2021). https://doi.org/10.1109/ICCVW54120.2021.00312.

Answer to Question 2:Thank you very much for your feedback. We have added an introduction to the latest developments in the SPP module in the [Introduction] section of the paper, including the evolution, structural ideas, and improvements of various versions of SPP. Additionally, in the [Improved SPP structure] section, we have provided a comprehensive explanation of our newly developed SPPFCSPC-G module.

Once again, we sincerely appreciate your valuable suggestions, as they have played a crucial role in improving our paper. We will continue to work diligently and incorporate your guidance to further enhance the content of the manuscript.

3.The experimental part needs to be richer. It is necessary to compare the method proposed in this paper with the existing detection methods, both qualitatively and quantitatively to illustrate the effectiveness of the proposed method.

Answer to Question 3:Thank you for your feedback. Based on your suggestions, we have made significant additions to the experimental section. We have included data from 13 different models, including VGG-16, Resnet-34, and various SPP modules. In this comparison, we have also incorporated SPP modules from YOLOv6 and YOLOv7 into YOLOv5 for experimentation. Ultimately, we have demonstrated the superior performance of the new SPPFCSPC-G module.

We have conducted both quantitative and qualitative comparisons to showcase the effectiveness of the proposed method in this paper. Our experimental results clearly indicate that the SPPFCSPC-G module outperforms other methods and effectively enhances the accuracy and efficiency of detection.

Once again, we express our gratitude for your valuable feedback. Your suggestions have played a pivotal role in improving the quality and content of our research.

4.Some figures in the experimental part are blurred, such as Fig. 4. It is recommended to increase the resolution of the image.

Answer to Question 4:Thank you for your feedback. We have re-created the figures in the experimental section and increased the resolution of the images. You will see these updated figures in the revised version of the paper.

Once again, we express our gratitude for your valuable suggestions. Your feedback has played a crucial role in improving the quality and presentation of our paper.

Reviewer #2: 

1.The change speed of YOLO series models is fast. Now YOLOv8 has been launched. Please elaborate why YOLOv5 is chosen as the main object detection model in this paper

Answer to Question 1:Thank you for your suggestion. (1) YOLOv5 has been widely used and studied in various real-world scenarios since its release in 2020. It has been widely accepted and researched by scholars worldwide. Researchers who study YOLO are familiar with its network structure and various improvements. Thus, YOLOv5 remains of great research value.

 (2) The network structure of YOLOv5 (BackBone, Neck, Head) is still considered a classic. Subsequent versions like YOLOv6/v7/v8 are based on the foundation of YOLOv5 with upgrades, such as adding attention mechanisms, improving loss functions, and enhancing modules like SPP, Conv, and CBL in the backbone. Decoupling mechanisms are also employed.

(3) YOLOv6 source code is mostly derived from YOLOv5, with certain components and structures improved. YOLOv7 maintains a similar overall structure to YOLOv5, using the backbone+FPN+PAN+head×3 format (consistent with YOLOv5). YOLOv8 also builds upon YOLOv5 and introduces new features like pose estimation and semantic segmentation. Therefore, YOLOv5 retains significant practical research value.

Once again, we appreciate your valuable feedback, and we believe YOLOv5's significance in our research remains substantial.

2. The innovation of the improved model in Section 2.3.1 is not enough. The core content is only replacing the original SPP module of YOLOv5

Answer to Question 2:Thank you for your feedback. In the [Improved SPP structure] section of the paper, we made significant innovations to the SPP module and introduced the SPPFCSPC-G module. We didn't simply replace the original SPP module in YOLOv5; instead, we conducted in-depth optimizations and improvements. Additionally, we experimented by incorporating the SPP modules from YOLOv6 and YOLOv7 into YOLOv5, and the results confirmed that our new SPPFCSPC-G module outperforms them.

We firmly believe that these improvements have made a significant contribution to enhancing the performance and accuracy of our proposed method in this paper. We appreciate your interest in our research and valuable feedback.

3. The experimental analysis part lacks the performance comparison with other classical single-stage and two-stage models, and the workload is insufficient

Answer to Question 3:Thank you for your feedback. We have included data from 13 different models, including VGG-16, Resnet-34, and various SPP modules. We also conducted comparative experiments with the SPP modules from YOLOv6 and YOLOv7, and tested them by integrating into YOLOv5. The results showed that our new SPPFCSPC-G module outperforms the others.

Once again, we sincerely appreciate your valuable feedback, and we have made the necessary revisions in the revised version of the paper.

4. The final recognition results show the recognition effect of the improved network model, but the number of recognition renderings is insufficient, which can not well reflect the performance improvement of the improved model

Answer to Question 4:Thank you for your valuable feedback.(1) Based on your suggestion, we have included more model performance parameters in the revised paper, which are presented in Table 1, Table 2, Table 3, and Table 4. Through these data, we have thoroughly discussed the advancements of our model, covering various aspects such as Precision, Recall, Loss, mAP, GPU-mem, Parameters, GFLOPs, Params., training speed, and inference speed. Specific data and results can be found in the new version of our submitted paper.

(2) In response to your recommendation, we have added more recognition result images in the paper, as shown in Figure 7. Through these additional recognition result images, we aim to better demonstrate the performance improvement of our improved model.

Once again, we appreciate your attention to our paper and your valuable feedback. Your suggestions have played a crucial role in enhancing the quality and presentation of our research. We have made the necessary modifications in the paper accordingly.

Reviewer #3: 

1.Authors should improve the organization of the Paper and the writing style.

Answer to Question 1:Thank you for your suggestion. We have made improvements to the writing style of the paper, aiming to make the new version more clear and readable. Once again, we appreciate your attention and valuable feedback on our paper.

2. The Introduction should cover problem definition, motivation and contributions.

Answer to Question 2:Thank you for your feedback. We have revised the Introduction section to include problem definition, motivation, and contributions. These additions aim to provide a more comprehensive and informative introduction to our research. Once again, we appreciate your valuable input on our paper.

3. The authors should add a seperate section for Related work and cover all the latest and relevant works such SE_SPNet and ktehr studies.

Answer to Question 3:Thank you for your feedback. We have added a new section in the Introduction, providing an overview and review of the latest developments in the SPP module. This includes the ideas and improvements proposed by scholars for various versions of the SPP structure. We hope these additions will better demonstrate the position and significance of our research in the context of existing work. Once again, we appreciate your attention to our paper and your valuable feedback.

4. Add a comparative table in the Retaled work section. Highlighting limitations of the existing works.

Answer to Question 4:Thank you for your feedback. We have included several models in our study, and in the revised version of the paper, we have provided comparative tables, specifically Table 1, Table 2, Table 3, and Table 4. These tables demonstrate the advanced performance of our model in various aspects, including Precision, Recall, Loss, mAP, GPU-mem, Parameters, GFLOPs, Params., training speed, and inference speed. For specific data, please refer to our newly submitted paper.

5. Please provide the rationale behind selection of the proposed model.

Answer to Question 5:Thank you for your feedback. In the revised version of the paper, we have provided a detailed description of the improvements made to the SPP module in the [Improved SPP structure] section. We have also included Figure 3, Figure 4, and Equation (1) in the revised paper to illustrate the rationale behind the selection of our proposed model. These additions aim to provide a clear and comprehensive explanation of why we chose this particular model for our research.

6. Discuss computational complexity of the proposed work and compare it with existing works.

Answer to Question 6:Thank you for your suggestion. In the newly submitted paper, you can find experimental data related to computational complexity parameters such as GPU-mem, Parameters, GFLOPs, and Params. in Table 3 and Table 4. We have also provided corresponding explanations to compare the computational complexity of our proposed work with existing works.

7. Compare your work with state of the art techniques and other similar works.

Answer to Question 7:Thank you for your feedback. We have included several models and provided comparative performance data in Table 1, which demonstrates the advanced performance of our model in terms of Precision, Recall, Loss, mAP, GPU-mem, Parameters, GFLOPs, Params., training speed, and inference speed. Please refer to the revised paper for specific data and detailed discussions. Once again, we appreciate your attention to our paper and your valuable feedback.

8. Discuss limitations of the proposed technique.

Answer to Question 8:Thank you for your feedback. In the [Conclusion] section of the paper, we have discussed the limitations of our technique. We address the issue of decreased inference speed caused by the new model and the significant performance variation of the optimizer on the dataset. Our research team will further explore and investigate these factors in future work.

Attachment Submitted filename: Response to Reviewers.docx Click here for additional data file.

# Peer review: Decision Letter 1

**DOI:** 10.1371/journal.pone.0295661.r003

**Authors:** Sunder Ali Khowaja

11 Sep 2023

PONE-D-23-12038R1Research on Rice Disease Recognition based on Improved SPPFCSPC-G YOLOv5 NetworkPLOS ONE

Dear Dr. Zhang,

Thank you for submitting your manuscript to PLOS ONE. After careful consideration, we feel that it has merit but does not fully meet PLOS ONE’s publication criteria as it currently stands. Therefore, we invite you to submit a revised version of the manuscript that addresses the points raised during the review process.

==============================

ACADEMIC EDITOR: Although the authors have revised the manuscript, the paper still have drawbacks in terms of rationality, literature review, and comparative analysis. Therefore, I would request authors to revise the manuscript according to the reviewer comments.

==============================

Please submit your revised manuscript by Oct 26 2023 11:59PM. If you will need more time than this to complete your revisions, please reply to this message or contact the journal office at plosone@plos.org. When you're ready to submit your revision, log on to https://www.editorialmanager.com/pone/ and select the 'Submissions Needing Revision' folder to locate your manuscript file.

Please include the following items when submitting your revised manuscript:

- A rebuttal letter that responds to each point raised by the academic editor and reviewer(s). You should upload this letter as a separate file labeled 'Response to Reviewers'.
- A marked-up copy of your manuscript that highlights changes made to the original version. You should upload this as a separate file labeled 'Revised Manuscript with Track Changes'.
- An unmarked version of your revised paper without tracked changes. You should upload this as a separate file labeled 'Manuscript'.

If you would like to make changes to your financial disclosure, please include your updated statement in your cover letter. Guidelines for resubmitting your figure files are available below the reviewer comments at the end of this letter.

If applicable, we recommend that you deposit your laboratory protocols in protocols.io to enhance the reproducibility of your results. Protocols.io assigns your protocol its own identifier (DOI) so that it can be cited independently in the future. For instructions see: https://journals.plos.org/plosone/s/submission-guidelines#loc-laboratory-protocols. Additionally, PLOS ONE offers an option for publishing peer-reviewed Lab Protocol articles, which describe protocols hosted on protocols.io. Read more information on sharing protocols at https://plos.org/protocols?utm_medium=editorial-email&utm_source=authorletters&utm_campaign=protocols.

We look forward to receiving your revised manuscript.

Kind regards,

Sunder Ali Khowaja, Ph.D.

Academic Editor

PLOS ONE

[Note: HTML markup is below. Please do not edit.]

Reviewers' comments:

Reviewer's Responses to Questions

**Comments to the Author**

1. If the authors have adequately addressed your comments raised in a previous round of review and you feel that this manuscript is now acceptable for publication, you may indicate that here to bypass the “Comments to the Author” section, enter your conflict of interest statement in the “Confidential to Editor” section, and submit your "Accept" recommendation.

Reviewer #1: (No Response)

Reviewer #4: (No Response)

**********

2. Is the manuscript technically sound, and do the data support the conclusions?

The manuscript must describe a technically sound piece of scientific research with data that supports the conclusions. Experiments must have been conducted rigorously, with appropriate controls, replication, and sample sizes. The conclusions must be drawn appropriately based on the data presented. 

Reviewer #1: Yes

Reviewer #4: No

**********

3. Has the statistical analysis been performed appropriately and rigorously? 

Reviewer #1: (No Response)

Reviewer #4: No

**********

4. Have the authors made all data underlying the findings in their manuscript fully available?

The PLOS Data policy requires authors to make all data underlying the findings described in their manuscript fully available without restriction, with rare exception (please refer to the Data Availability Statement in the manuscript PDF file). The data should be provided as part of the manuscript or its supporting information, or deposited to a public repository. For example, in addition to summary statistics, the data points behind means, medians and variance measures should be available. If there are restrictions on publicly sharing data—e.g. participant privacy or use of data from a third party—those must be specified.

Reviewer #1: (No Response)

Reviewer #4: Yes

**********

5. Is the manuscript presented in an intelligible fashion and written in standard English?

PLOS ONE does not copyedit accepted manuscripts, so the language in submitted articles must be clear, correct, and unambiguous. Any typographical or grammatical errors should be corrected at revision, so please note any specific errors here.

Reviewer #1: (No Response)

Reviewer #4: Yes

**********

6. Review Comments to the Author

Please use the space provided to explain your answers to the questions above. You may also include additional comments for the author, including concerns about dual publication, research ethics, or publication ethics. (Please upload your review as an attachment if it exceeds 20,000 characters)

Reviewer #1: The questions have been addressed well. I think this paper can be published in the PLOS ONE Journal.

Reviewer #4: The authors tried to address all reviewer's comments/feedbacks. However, there are lacks of clarity and answers are not fully convincing.

However, the experimental strategy/methodology and proof-of-correctness is questionable.

1. First of all, to demonstrate the effectiveness of proposed approach authors must compare with previous research work(s) on same dataset, say previous few architectures/improved architectures on one/more same dataset (in this work, rice-disease dataset). Authors should demonstrate the improvement against similar works.

2. What is the motivation against selecting VGG-16 and/or ResNet-34, why not other VGG-x/ResNet-x architectures ? Even, baseline Yolov-x recent architectures also have been modified. Please demonstrate a comparison against Recent YOLOv-x architectures as well like, utilizing SAHI , Yolov8, Yolo-NAS or ViT based models. Authors should not use an arbitrary choice of models to demonstrate improvement. Also, if improvement metric should be considered with previous modules , those are not convincing at all (as authors only proposed improvement on a particular dataset or use case, whereas baseline models generally benchmarked on an universal dataset like PASCAL/COCO etc.). The improvement factors to be noticed as .50% against SimSPPF[17] or 1.0% against SPPFCSPC only for precision, 1.1% or 1.3% for recall for same models, +1.2% against SPPCSPC[18] for mAP @0.5 and 0.8% for map @0.95..There is surely no clear trend of improvement to be followed as previous baseline models are also constantly evolving and showing improvement against each other. Therefore, how significant is authors proposed method only on a particular dataset? As authors are mainly claiming improvement of proposed module, please demonstrate the generalizability on a very different public dataset (may be a different domain if possible, like occluded object detection/satellite imagery object detection etc. )

3. The citations in Table 1is misleading to readers as [16][17][18][20][21] are not directly related to rise-disease detection application and mostly baseline models. Readers may think authors compared with previous related research works (which should be more relevant).

I do not recommend this manuscript to be accepted in its present version. The experimental methodology and results are not convincing and not justifying towards demonstrating any novelty/uniqueness to extended research community.

**********

7. PLOS authors have the option to publish the peer review history of their article (what does this mean?). If published, this will include your full peer review and any attached files.

If you choose “no”, your identity will remain anonymous but your review may still be made public.

**Do you want your identity to be public for this peer review?** For information about this choice, including consent withdrawal, please see our Privacy Policy.

Reviewer #1: No

Reviewer #4: No

**********

[NOTE: If reviewer comments were submitted as an attachment file, they will be attached to this email and accessible via the submission site. Please log into your account, locate the manuscript record, and check for the action link "View Attachments". If this link does not appear, there are no attachment files.]

While revising your submission, please upload your figure files to the Preflight Analysis and Conversion Engine (PACE) digital diagnostic tool, https://pacev2.apexcovantage.com/. PACE helps ensure that figures meet PLOS requirements. To use PACE, you must first register as a user. Registration is free. Then, login and navigate to the UPLOAD tab, where you will find detailed instructions on how to use the tool. If you encounter any issues or have any questions when using PACE, please email PLOS at figures@plos.org. Please note that Supporting Information files do not need this step.

# Author comment: Author response to Decision Letter 1

**DOI:** 10.1371/journal.pone.0295661.r004

13 Oct 2023

Dear esteemed journal editor and reviewers

Firstly, we would like to sincerely thank you for reviewing our paper and providing valuable feedback. Your professional opinions and suggestions have been of great help to our research.

In our response, we have addressed each question raised by the reviewers and made necessary revisions and improvements based on your suggestions. The following is the work we have done in response to each question:

Reviewer #4: 

Question 1: First of all, to demonstrate the effectiveness of proposed approach authors must compare with previous research work(s) on same dataset, say previous few architectures/improved architectures on one/more same dataset (in this work, rice-disease dataset). Authors should demonstrate the improvement against similar works.

Answer to Question 1:Thank you for your valuable feedback. Our research work is carried out in conjunction with our fund project. Our research direction is mainly to use computer vision technology to identify and detect the disease of rice in specific scenarios.

In the popular COCO, ImageNet, and PASCAL datasets, the objects in each image are not rice (our experimental research object is rice). Although there are some rice objects in the COCO dataset, there are also other objects in each image, and their quantity is huge. Although such public datasets are good for verifying the generalization ability of models, our work can only perform image recognition on a specific rice dataset. Therefore, by training a dedicated rice dataset, we can obtain model parameters and prediction models that are more in line with actual conditions, thereby improving the targeting and accuracy of the model.

Question 2：What is the motivation against selecting VGG-16 and/or ResNet-34, why not other VGG-x/ResNet-x architectures ?[2-1] Even, baseline Yolov-x recent architectures also have been modified. Please demonstrate a comparison against Recent YOLOv-x architectures as well like, utilizing SAHI , Yolov8, Yolo-NAS or ViT based models. Authors should not use an arbitrary choice of models to demonstrate improvement. [2-2]Also, if improvement metric should be considered with previous modules , those are not convincing at all (as authors only proposed improvement on a particular dataset or use case, whereas baseline models generally benchmarked on an universal dataset like PASCAL/COCO etc.). [2-3]The improvement factors to be noticed as .50% against SimSPPF[17] or 1.0% against SPPFCSPC only for precision, 1.1% or 1.3% for recall for same models, +1.2% against SPPCSPC[18] for mAP @0.5 and 0.8% for map @0.95..There is surely no clear trend of improvement to be followed as previous baseline models are also constantly evolving and showing improvement against each other. Therefore, how significant is authors proposed method only on a particular dataset? As authors are mainly claiming improvement of proposed module, please demonstrate the generalizability on a very different public dataset (may be a different domain if possible, like occluded object detection/satellite imagery object detection etc. )[2-4]

Answer to Question 2:Thank you for your valuable feedback. Regarding Question 2, we will answer it in four parts.

Answer to Question 2-1: In Table 1, we replaced three additional experiments. At the same time, we replaced the original SPPF module with the SPPCSPC-G module designed in this experiment.

Our research work is mainly focused on lightweight models and practical applications, and the trained models will be applied to mobile devices.

Although deeper convolutional neural network structures such as VGG-16/19/30, ResNet-50/101/152, RepVGG-A0/A1/B0/B1, ViTAE-T/S/v2-S represent stronger expression ability and more complex feature learning ability, they may also provide higher computational costs and more difficult-to-train models, with low computational efficiency. The computational resource consumption of deeper network architectures is very large, and the model training time is also very long. At the same time, because of the deep architecture, the models trained are often very large. If a target detection model is deployed on a mobile device, a model that is too large may cause some problems. For example: (1) The storage space of mobile devices is limited, and too large models may occupy too much storage space, thereby affecting the download and use experience of applications. (2) The computing power of mobile devices is relatively limited, and too much computing power may affect the response speed and battery life of applications. The computational cost of target detection models is usually large. If deployed on a mobile device, it may put great pressure on the performance of mobile devices.

In addition, our research work uses computer vision technology to identify and detect the disease of rice in specific scenarios. We do not develop a large network architecture to adapt to different scenarios or different complexity tasks (fine-grained classification, small object detection, multi-object detection, etc.). Our project requirements do not need deeper models. When choosing a suitable model architecture, the project team needs to weigh according to specific tasks and computing resources, without blindly pursuing deep architectures. Therefore we did not choose other VGG-x/ResNet-x architectures.

Answer to Question 2-2: (1) First of all, the performance difference between Yolov5 and YOLOv8/v7/v6-3.0 on the COCO dataset is not very significant, as shown in the following figure.

(2) Second, our research work mainly improved one of the SPP modules. Therefore, we used the same type of SPP module as the comparison module (which also includes the SPP module in the latest YOLOv7), and then conducted experiments and verification under the YOLOv5 framework. The final training results are shown in the following table. Precision, recall, and mAP values are very high on our dataset, so the project team considers using YOLOv8/v7 to verify our SPPCSPC-G module on the rice dataset, and it is difficult to further improve performance.

Framework Model Optimizer SteadyEpoch Precision Recall 

YOLOv5-s SPPFCSPC-G(Our) SGD 200 96.20% 99.2% 98.60% 86.80%

Answer to Question 2-3: Our research work is based on our experimental fund projects (1) (2) (3). Our research direction is mainly to use computer vision technology to identify and detect the disease of rice in specific scenarios.

Therefore, the project team established a rice disease dataset to solve the problem of insufficient traditional data. Although traditional generalized datasets (COCO/Imagenet) contain a large amount of data, they cannot provide sufficient data on rice growth and breeding. Therefore, by training a dedicated rice dataset, the problem of insufficient data in the rice field can be solved, and the training effect and prediction accuracy of the model can be improved. Since the growth environment and growth situation of rice are relatively complex, more refined data is needed to support model training and prediction. Training a dedicated rice dataset can provide more refined data, including various details and features during rice growth, thereby better supporting model training and prediction.

(1) Research project on intelligent monitoring and early warning system for rice diseases and insect pests based on meteorological conditions

(2) Intelligent agricultural trusted tracking system based on blockchain

(3) Key technology research on corn seed selection based on convolutional neural network

Answer to Question 2-4: We will show the models trained on the COCO dataset at different network depths of YOLOv5-s/x/l to identify rice disease leaves. As shown in the figure below, the models trained on the COCO dataset did not accurately identify and detect the research object for rice leaves using YOLOv5-s/x. Although YOLOv5-l recognized the object in the first two figures, almost all of them were incorrect. Therefore, this is why the project team needs to train on a specific dataset.

Original drawing

 YOLOv5-S

 YOLOv5-X

 YOLOv5-L

Although training on public datasets can detect the model’s generalization ability, our project fund’s research objects are only rice and corn. Therefore, the project team did not show the generalization ability of the SPPFCSPC-G module on public datasets in the paper.

Question 3：The citations in Table 1is misleading to readers as [16][17][18][20][21] are not directly related to rise-disease detection application and mostly baseline models. Readers may think authors compared with previous related research works (which should be more relevant).

Answer to Question 3: Thank you for your valuable feedback. We have removed the unreasonable reference identifiers in Table 1 and revised and improved Table 3 to make it more standardized.

Thank you again for your valuable feedback.

Attachment Submitted filename: Response to Reviewers.docx Click here for additional data file.

# Peer review: Decision Letter 2

**DOI:** 10.1371/journal.pone.0295661.r005

**Authors:** Sunder Ali Khowaja

28 Nov 2023

Research on Rice Disease Recognition based on Improved SPPFCSPC-G YOLOv5 Network

PONE-D-23-12038R2

Dear Dr. Zhang,

We’re pleased to inform you that your manuscript has been judged scientifically suitable for publication and will be formally accepted for publication once it meets all outstanding technical requirements.

Within one week, you’ll receive an e-mail detailing the required amendments. When these have been addressed, you’ll receive a formal acceptance letter and your manuscript will be scheduled for publication.

An invoice for payment will follow shortly after the formal acceptance. To ensure an efficient process, please log into Editorial Manager at http://www.editorialmanager.com/pone/, click the 'Update My Information' link at the top of the page, and double check that your user information is up-to-date. If you have any billing related questions, please contact our Author Billing department directly at authorbilling@plos.org.

If your institution or institutions have a press office, please notify them about your upcoming paper to help maximize its impact. If they’ll be preparing press materials, please inform our press team as soon as possible -- no later than 48 hours after receiving the formal acceptance. Your manuscript will remain under strict press embargo until 2 pm Eastern Time on the date of publication. For more information, please contact onepress@plos.org.

Kind regards,

Sunder Ali Khowaja, Ph.D.

Academic Editor

PLOS ONE

Additional Editor Comments (optional):

The reviewers have completed their reviews. They found the revisions to be satisfactory and adequate. Therefore, I would like to recommend the acceptance of this article.

Reviewers' comments:

Reviewer's Responses to Questions

**Comments to the Author**

1. If the authors have adequately addressed your comments raised in a previous round of review and you feel that this manuscript is now acceptable for publication, you may indicate that here to bypass the “Comments to the Author” section, enter your conflict of interest statement in the “Confidential to Editor” section, and submit your "Accept" recommendation.

Reviewer #1: (No Response)

Reviewer #5: All comments have been addressed

**********

2. Is the manuscript technically sound, and do the data support the conclusions?

The manuscript must describe a technically sound piece of scientific research with data that supports the conclusions. Experiments must have been conducted rigorously, with appropriate controls, replication, and sample sizes. The conclusions must be drawn appropriately based on the data presented. 

Reviewer #1: (No Response)

Reviewer #5: Yes

**********

3. Has the statistical analysis been performed appropriately and rigorously? 

Reviewer #1: (No Response)

Reviewer #5: N/A

**********

4. Have the authors made all data underlying the findings in their manuscript fully available?

The PLOS Data policy requires authors to make all data underlying the findings described in their manuscript fully available without restriction, with rare exception (please refer to the Data Availability Statement in the manuscript PDF file). The data should be provided as part of the manuscript or its supporting information, or deposited to a public repository. For example, in addition to summary statistics, the data points behind means, medians and variance measures should be available. If there are restrictions on publicly sharing data—e.g. participant privacy or use of data from a third party—those must be specified.

Reviewer #1: (No Response)

Reviewer #5: No

**********

5. Is the manuscript presented in an intelligible fashion and written in standard English?

PLOS ONE does not copyedit accepted manuscripts, so the language in submitted articles must be clear, correct, and unambiguous. Any typographical or grammatical errors should be corrected at revision, so please note any specific errors here.

Reviewer #1: (No Response)

Reviewer #5: Yes

**********

6. Review Comments to the Author

Please use the space provided to explain your answers to the questions above. You may also include additional comments for the author, including concerns about dual publication, research ethics, or publication ethics. (Please upload your review as an attachment if it exceeds 20,000 characters)

Reviewer #1: (No Response)

Reviewer #5: Authors have addressed all the comments in a satisfactory manner. Therefore, I would like to recommend the acceptance of this article.

**********

7. PLOS authors have the option to publish the peer review history of their article (what does this mean?). If published, this will include your full peer review and any attached files.

If you choose “no”, your identity will remain anonymous but your review may still be made public.

**Do you want your identity to be public for this peer review?** For information about this choice, including consent withdrawal, please see our Privacy Policy.

Reviewer #1: No

Reviewer #5: **Yes: **Parus Khuwaja

**********

# Editor report: Acceptance letter

**DOI:** 10.1371/journal.pone.0295661.r006

**Authors:** Sunder Ali Khowaja

6 Dec 2023

PONE-D-23-12038R2 

Research on Rice Disease Recognition based on Improved SPPFCSPC-G YOLOv5 Network 

Dear Dr. Zhang:

I'm pleased to inform you that your manuscript has been deemed suitable for publication in PLOS ONE. Congratulations! Your manuscript is now with our production department. 

If your institution or institutions have a press office, please let them know about your upcoming paper now to help maximize its impact. If they'll be preparing press materials, please inform our press team within the next 48 hours. Your manuscript will remain under strict press embargo until 2 pm Eastern Time on the date of publication. For more information please contact onepress@plos.org.

If we can help with anything else, please email us at customercare@plos.org.

Thank you for submitting your work to PLOS ONE and supporting open access. 

Kind regards, 

PLOS ONE Editorial Office Staff

on behalf of

Dr. Sunder Ali Khowaja 

Academic Editor

PLOS ONE
